# Spin Filtering in Supramolecular Polymers Assembled from Achiral Monomers Mediated by Chiral Solvents

**DOI:** 10.1021/jacs.1c02983

**Published:** 2021-04-30

**Authors:** Amit Kumar Mondal, Marco D. Preuss, Marcin L. Ślęczkowski, Tapan Kumar Das, Ghislaine Vantomme, E. W. Meijer, Ron Naaman

**Affiliations:** † Department of Chemical and Biological Physics, Weizmann Institute of Science, Rehovot 76100, Israel; ‡ Institute for Complex Molecular Systems and Laboratory of Macromolecular and Organic Chemistry, Eindhoven University of Technology, P.O. Box 513, 5600 MB Eindhoven, The Netherlands

## Abstract

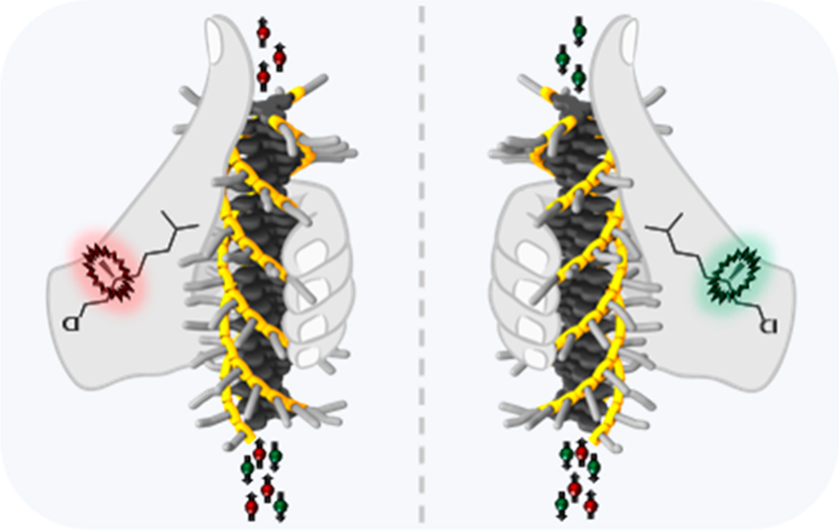

In past studies, spin selective transport was observed in polymers and supramolecular structures that are based on homochiral building blocks possessing stereocenters. Here we address the question to what extent chiral building blocks are required for observing the chiral induced spin selectivity (CISS) effect. We demonstrate the CISS effect in supramolecular polymers exclusively containing achiral monomers, where the supramolecular chirality was induced by chiral solvents that were removed from the fibers before measuring. Spin-selective transport was observed for electrons transmitted perpendicular to the fibers’ long axis. The spin polarization correlates with the intensity of the CD spectra of the polymers, indicating that the effect is nonlocal. It is found that the spin polarization increases with the samples’ thickness and the thickness dependence is the result of at least two mechanisms: the first is the CISS effect, and the second reduces the spin polarization due to scattering. Temperature dependence studies provide the first support for theoretical work that suggested that phonons may contribute to the spin polarization.

## Introduction

In recent years, it was established that electron transport through homochiral molecules depends on the electron spin. This phenomenon, named Chiral Induced Spin Selectivity (CISS) effect, means that chiral molecules can serve as efficient spin filters.^1^ During the past years, the CISS effect has been explored in biomolecules like DNA,^2^ oligopeptides,^3^ proteins,^4^ polymers,^5^ as well as in chiral perovskites,^6−8^ chiral supramolecular structures,^9^ and other molecules. In all cases, the materials are based on chiral building blocks with specific configurations at the stereocenters and exciting results were obtained with chiral supramolecular polymers.

In supramolecular polymerization processes of chiral molecules, the chiral information on the monomer is often translated to the secondary structural motif of the supramolecular polymer.^10^ The formation of one-dimensional, helical polymers is one of the commonly found motifs.^11−15^ Depending on the enantiomer of the monomer used and its enantiomeric excess, the polymers usually assemble with either an excess of right- (*P*) or left-handed (*M*) helical screw sense. With achiral monomers, a racemic mixture of *P-* and *M-*helices is observed.^10,12^ However, next to chiral monomers, supramolecular (homo)chirality has also been induced by other external stimuli such as mechanical stress,^16−21^ illumination with circularly polarized light,^22−25^ or chiral solvents.^12,26−28^ In cooperative systems,^29^ through which small energy differences accumulate into large effects, minor differences in solvation energy between *P* and *M* helical aggregates in chiral solvents can drive the supramolecular aggregate of achiral monomers to favor one helicity over the other.^27^ The secondary structural motif of the supramolecular polymer can adapt opposite helicity depending on the enantiomer of the chiral solvent used.^12,26^ Moreover, with kinetically stable supramolecular polymers, the chiral solvent can be removed, while the preferred helicity remains yielding a chiral superstructure without any chiral building block. Similar effects of the transfer of asymmetry by chiral solvents have been exploited to bias the secondary structural motif of nanotubes^30^ and polymers,^31,32^ or to enrich the enantiomeric excess in asymmetric synthesis.^33^ These recent findings allow discovering whether spin selection can be obtained solely due to chiral stacking of otherwise achiral building blocks.

There are numerous methods for monitoring spin selective transport through molecules; we rely mainly on magnetic-conductive atomic force microscopy (mc-AFM).^34^ The spin polarization at a specific electric potential difference, *V*, is expressed by *P*(*V*) = {[(*I*_down_(*V*) – *I*_up_(*V*))/(*I*_down_(*V*) + *I*_up_(*V*))] × 100}, when *I*_up_ and *I*_down_ are the currents measured at a given potential for the magnetic electrode magnetized with the north pole pointing towards the surface or away from its surface, respectively. For temperature-dependent spin transport studies, a device was fabricated, and the magnetoresistance was measured.^35^ Recent results on spin transport through chiral supramolecular polymers showed spin polarization values of up to 90%.^9^ The measurements were performed on polymers obtained from chiral monomers and from mixtures of chiral and achiral monomers following the “sergeant-and-soldiers-principle”.^36^ In the latter, a small quantity of chiral monomers (sergeant) mixed with achiral monomers (soldier) dictates the net helicity of the stacks. A direct correlation between net helicity and spin polarization has been observed, proving that the CISS effect is closely related to supramolecular chirality, and not only to the number of stereocenters.^9^ However, the question remains whether the presence of stereocenters in the helical structure is critical to the observation of spin polarization.

**Figure 1:**
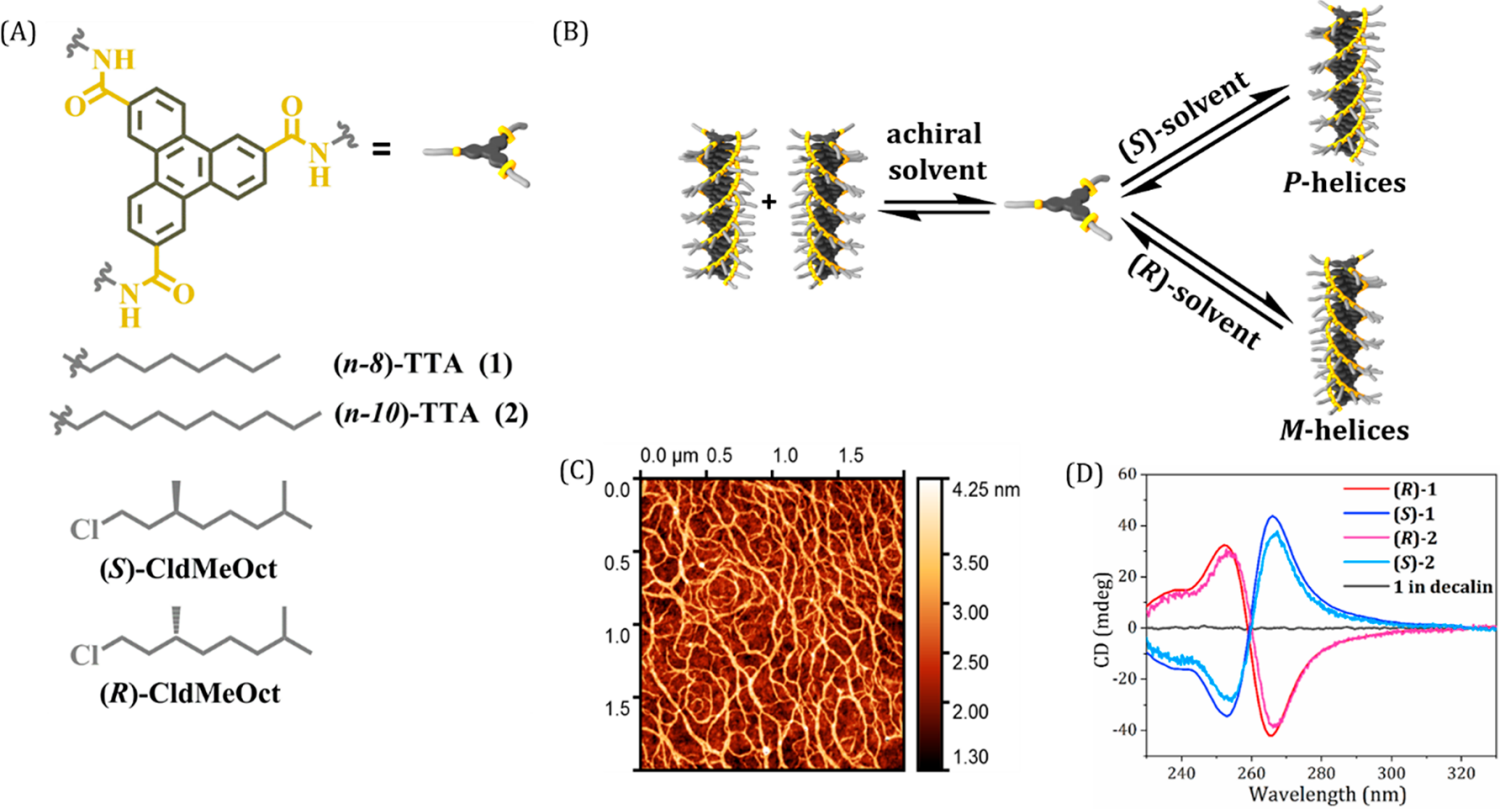
Molecules and the chiral solvents used. (A) Chemical structure of the molecules **1** and **2** and the chiral solvents (*S*)-CldMeOct and (*R*)-CldMeOct used in this study. (B) Schematic representation of the solvent mediated chiral self-assembled nanofibers formed with either *P-* or *M*-helicity. (C) High-resolution AFM image of the supramolecular structures obtained from **(*****S*****)-1**, drop-casted on silicon. (D) CD spectra of **1** and **2** in (*S*)-CldMeOct, (*R*)-CldMeOct, and decalin (achiral) (*c* = 75 μM, *d* = 1 mm, 20 °C).

In the present work, we demonstrate a case in which the CISS effect does not originate from the chirality of the monomers but is solely the result of the helical arrangement of monomers in supramolecular polymers. We report on spin-dependent transport through chiral aggregates of exclusively achiral monomers, assembled into chiral supramolecular polymers using chiral solvents. The study focuses on triphenylene-2,6,10-tricarboxamide (TTA) based supramolecular polymers with either octyl or decyl aliphatic side chains ((*n-8*)- and (*n-10*)-TTA, molecule **1** and **2**). These achiral monomers have been shown to assemble cooperatively into one-dimensional (1D) aggregates, with their supramolecular helicity biased by chiral chlorinated solvents (Figure 1).^12^ The study shows that both “up” and “down” orientations of filtered spins can be obtained with the same achiral monomer by drop-casting the samples from enantiomerically pure solvent, followed by removing the solvent. In addition, we report on a CISS-based magnetoresistance device that provides insight on the temperature dependence of the spin transport.

## Results and Discussion

The chiral polymers were first analyzed spectroscopically in (*S*)- and (*R*)-1-chloro-3,7-dimethyloctane (**(*****S*****)/(*****R*****)**) solution (see Supporting Information for synthesis and sample preparation). The supramolecular chirality of the fibers was monitored using circular dichroism (CD) spectroscopy. The monomers **1** and **2** were dissolved in both enantiomers of the chiral solvent **(*****S*****)** and **(*****R*****)** to give the monomer–solvent mixtures **(*****S*****)-1**, **(*****R*****)-1**, **(*****S*****)-2**, and **(*****R*****)-2**. In chiral solvent **(*****S*****)**, the achiral monomers cooperatively assemble into supramolecular polymers and display a positive Cotton effect with a maximum at 266 nm at room temperature. In chiral solvent **(*****R*****)**, a mirror image Cotton effect of opposite sign is obtained by cooperative assembly, indicating the helical inversion of the screw sense of the polymers in the enantiomeric solvent. The mirror symmetry of the CD spectra of **(*****S*****)-1/2** and **(*****R*****)-1/2** suggests an enantiomeric nature of the supramolecular polymers (Figure 1D). Slight changes in the intensity of the CD signal can be observed between polymers **(*****S*****)/(*****R*****)-1** and **(*****S*****)/(*****R*****)-2** (Figure 1D). Polymer **2** shows a lower CD intensity compared to **1**, suggesting a small difference in the efficacy of transfer of asymmetry from the solvent to the supramolecular stacks of **2**. This could be a result of solvation differences caused by the different lengths of the aliphatic side chains of the monomers. In achiral solvent such as decalin, no preferred helicity was observed (Figure 1D). By spin-coating **1** from (*S*)-1-chloro-2-methylbutane onto quartz glass, it was shown that the supramolecular polymers are able to maintain their preferred helical orientation in bulk (SI, Figure S10). With drying the samples for multiple hours under ambient conditions, it is assumed that all chiral solvent is removed, but it cannot be excluded that residual amounts of chiral solvent remain in the films.

**Figure 2:**
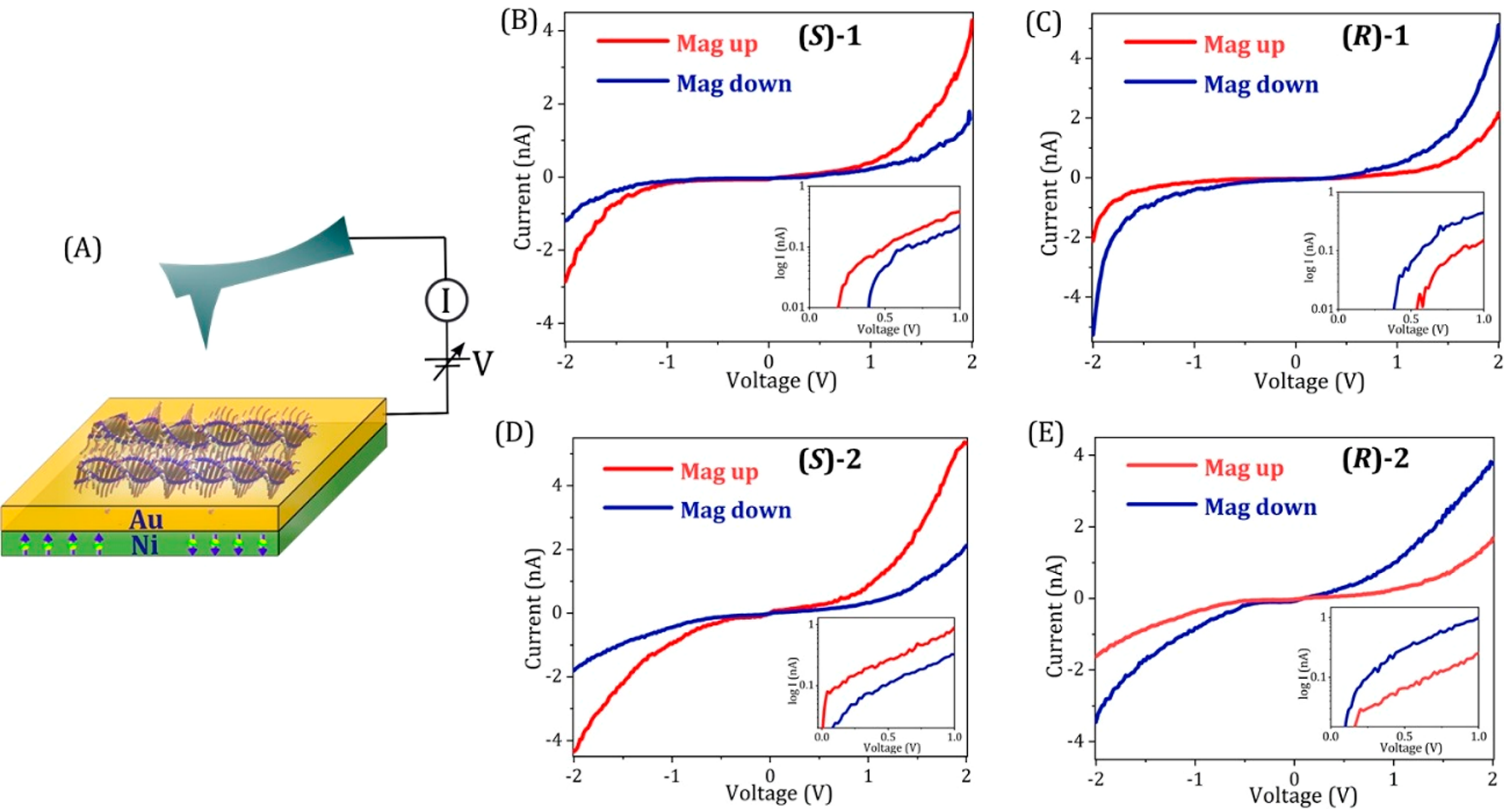
Spin polarization measured with the mc-AFM. (A) Schematic representation of the magnetic-conductive atomic force microscopy (mc-AFM) setup. Panels B and C present the averaged current versus voltage (*I*–*V*) curves recorded for **(*****S*****)-1** and **(*****R*****)-1**, respectively, with the magnet north pole pointing up (red) or down (blue). Panels D and E show the averaged *I*–*V* curves for **(*****S*****)-2** and **(*****R*****)-2**, respectively. (Inset) Corresponding curves as a semi log plot. The sample thickness in all the measurements was 5 ± 1 nm.

Magnetic conducting atomic force microscopy (mc-AFM) measurements were performed to study the spin selectivity of electron transmission through all the TTA polymers. A scheme representing the mc-AFM setup is shown in Figure 2A. The supramolecular nanofibers are formed using **(*****S*****)-1/2** and **(*****R*****)-1/2** and drop-casted on a gold coated nickel surface (Au/Ni), that is magnetized with its magnetization perpendicular to the surface with the north-pole pointing either up or down, followed by drying for few hours in an oven. The AFM tip is maintained at ground while the potential on the Au/Ni is changed. The current measurements are performed in a perpendicular setup respective to the long axis of the fiber. However, it is expected that the current will pass the fibers starting from a circular area underneath the tip, following the most conducting path. Hence, some of the electrons will follow partly the direction of the fibers’ long axis. As shown in Figure 2B-E the current changes significantly when the magnetic electrode was magnetized UP or DOWN. Each plot is the average of about 40–50 I vs V scans.

**Figure 3:**
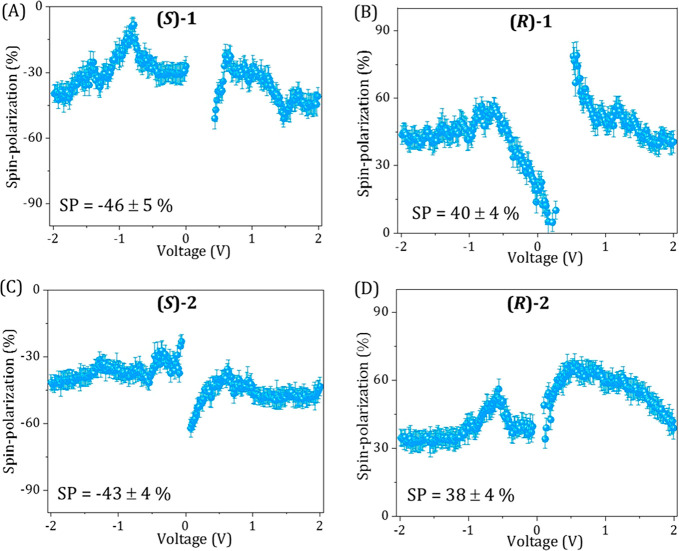
Spin polarization calculated based on the results shown in Figure 2. (A, B) Spin polarization as a function of applied bias for **(*****S*****)-1** and **(*****R*****)-1**, respectively. (C, D) Spin polarization as a function of applied bias for **(*****S*****)-2** and **(*****R*****)-2**, respectively. The percentage of spin polarization is calculated as {[(*I*_down_ – *I*_up_)/(*I*_down_ + *I*_up_)] × 100}. *I*_up_ and *I*_down_ are the currents measured with the magnetic north pole pointing up and down, respectively.

In the case of the fibers formed in **(*****S*****)**-solvent, higher current is observed when the electrons are injected from the substrate magnetized UP (Figure 2B, D). The opposite behavior is observed for the fibers formed in **(*****R*****)**-solvent, where current is higher when the substrate is magnetized in the DOWN direction (Figure 2C, E). All the plots observed result from nonlinear dependence of the current on the voltage applied. The corresponding semilog plots, that are presented in the insets, demonstrate the nonlinearity of the process. Clearly two different onsets of the currents are observed, each corresponds to a different spin polarization. The distinct threshold for each spin indicates that no spin flipping occurs during the conduction process. In the case of films processed from **(*****R*****)** the lower threshold corresponds to the magnet pointing down, i.e. the spins injected are polarized parallel to the electron’s velocity and the opposite is true for fibers processed from **(*****S*****)**. To prove the role of the chiral solvent in determining the supramolecular chirality and thus the spin filtering, we performed control measurements for fibers of **1** assembled in the achiral solvent decalin. In this case, there is no difference in the *I* vs *V* curves when the magnetic field was directed UP or DOWN (SI and Figure S1), suggesting no spin selective transport through racemic mixtures of supramolecular polymers. The calculated spin polarization, based on the results shown in Figure 2, is presented in Figure 3.

The spin polarization for all four cases studied, **(*****R*****/*****S*****)**-**1** and **(*****R*****/*****S*****)-2**, is about 40%. Please note that while in the case of the fibers formed in **(*****S*****)**-solvent, the polarization has a negative sign; it is positive for fibers made in **(*****R*****)**-solvent. At very low voltage, since there are different thresholds for each spin, the polarization is very high. The origin for the fluctuations in the polarization at higher potential are not clear and may result from variations in the thickness of the fiber bundles when each thickness corresponds to different extend of polarization. The superposition of curves obtained with fiber bundles of different thicknesses may result in nonmonotonic curves. Moreover, as pointed out above, the transfer of asymmetry from the chiral solvent to the polymers **1** and **2** results in slight differences in their CD intensities (Figure 1D). Although within the error of the measurements, this difference coincides with the spin polarization of **2** being slightly lower than the spin polarization observed for **1** (Figure 3).

**Figure 4:**
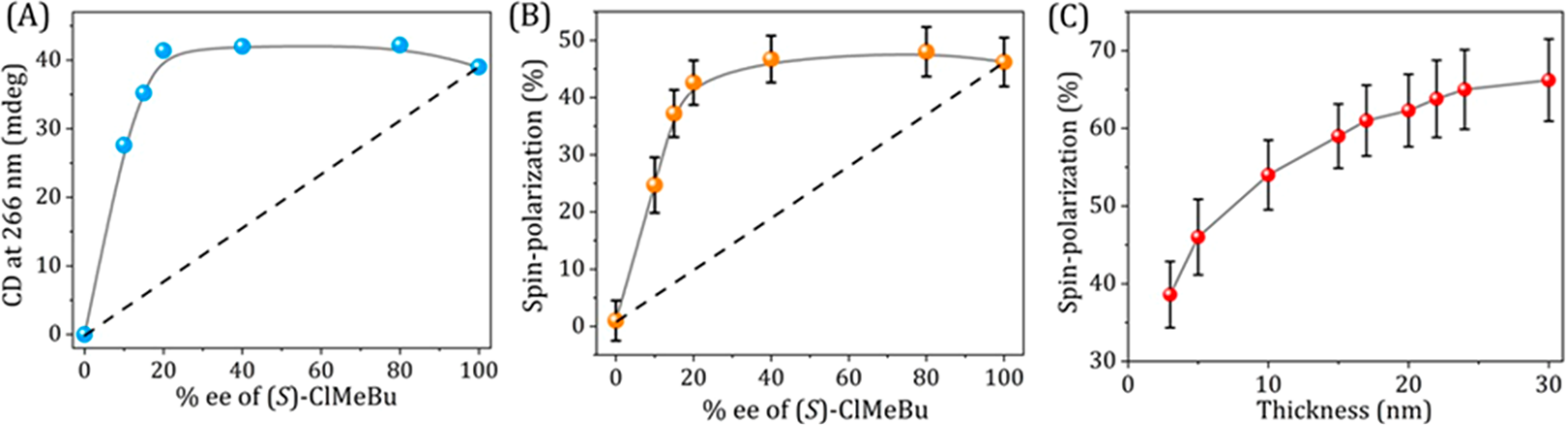
Effect of *ee* of the solvent on the CD and spin polarization and the thickness dependent spin polarization. (A) The intensity of the CD at 266 nm of **1**, as a function of the *ee* of the (*S*)-ClMeBu solvent. The nonlinear dependence of CD signal indicates amplification of asymmetry of the solvent chirality in the assembly processes. (B) Absolute value of spin polarization at +2 V measured using mc-AFM-based *I*–*V* curves at different *ee* of the (*S*)-ClMeBu solvent. The same solutions were used for the CD studies and for the formation of the nanofibers. All the measurements were conducted for sample thickness of 5 ± 1 nm. The solid-gray lines in (A) and (B) are to guide the eye. The dashed black line suggests the expected trend in the absence of amplification of asymmetry. (C) Dependence of the spin polarization (absolute value) of **(*****S*****)-1** on the thickness of the layers. See the text for details on the thickness dependence studies.

Intrigued by this result, we further aimed to correlate the net helicity of the supramolecular stacks with the observed spin polarization. As changing the chain length of the aliphatic side chains only resulted in minor differences of net helicity, we aimed to tune the net helicity of the supramolecular stacks of **1** by changing the enantiomeric excess (*ee*) of the solvent. To obtain solvent mixtures of different *ee*, enantiopure (*S*)-1-chloro-2-methylbutane ((*S*)-ClMeBu) was mixed with commercially available racemic ClMeBu. As shown in Figure 4A, in a racemic mixture of (*R*)- and (*S*)-ClMeBu (0% *ee*), no CD signal and no net helicity could be observed. Upon increasing the *ee* of ClMeBu mixtures to 20%, the CD intensity, monitored at 266 nm, increases nonlinearly, due to a majority-rules effect. At higher *ee*, the CD signal saturates to 40 mdeg. A similar behavior is found here in the measured spin polarization as determined at a voltage of 2 V (Figure 4B). All the results are given for a sample thickness of 5 ± 1 nm. The spin polarization saturates as the CD signal does. Hence, there is a direct correlation between the *ee* of the solvent, the CD signal of the polymers, and the spin-polarization measured by mc-AFM.

The layers of the supramolecular fibers are formed with variability in their thickness. Since the spin polarization is measured perpendicular to the fibers’ long axis, it is expected that its values will depend on the thickness of the samples, resulting from clustering of multiple fibers together. The thickness dependence measurements were performed with fibers prepared from (*S*)-CldMeOct solvent. Samples of varied thicknesses between 3 ± 1 nm and 30 ± 1 nm were made by increasing the sample quantity on the surface by multiple drop-casting steps. Before performing each *I* vs *V* measurement, the thickness of the samples was measured by AFM. A thickness dependence of the spin polarization was indeed found, as shown in Figure 4C. However, interestingly even though the thickness is varied by a factor of 10, the spin polarization increases by not more than 30%. In the case of DNA and oligopeptides monolayers, it was found that the spin polarization scales about linearly with the thickness of the monolayer, up to about 12 nm.^37^ Interestingly, also in Figure 4C, the slope of the dependence of the polarization on the thickness changes at about the same thickness of about 15 nm. This change in the slope may indicate that for samples above 15 nm in thickness an additional mechanism becomes significant. Hence, the weak dependency on the thickness observed here, for large thicknesses, may result from two competing mechanisms. The first is the regular CISS effect, which tends to increase about linearly with the molecular length, and the second is the spin depolarization due to collisions within the fibers. This spin depolarization seems to become significant at thicknesses of about 15 nm. The recently observed spin polarization as measured in spin transport through thick systems, where the thickness reaches even hundreds of nanometers,^6,38^ may also be explained by the existence of these two mechanisms: the first keeps inducing spin polarization, and the other causes its decay.

**Figure 5:**
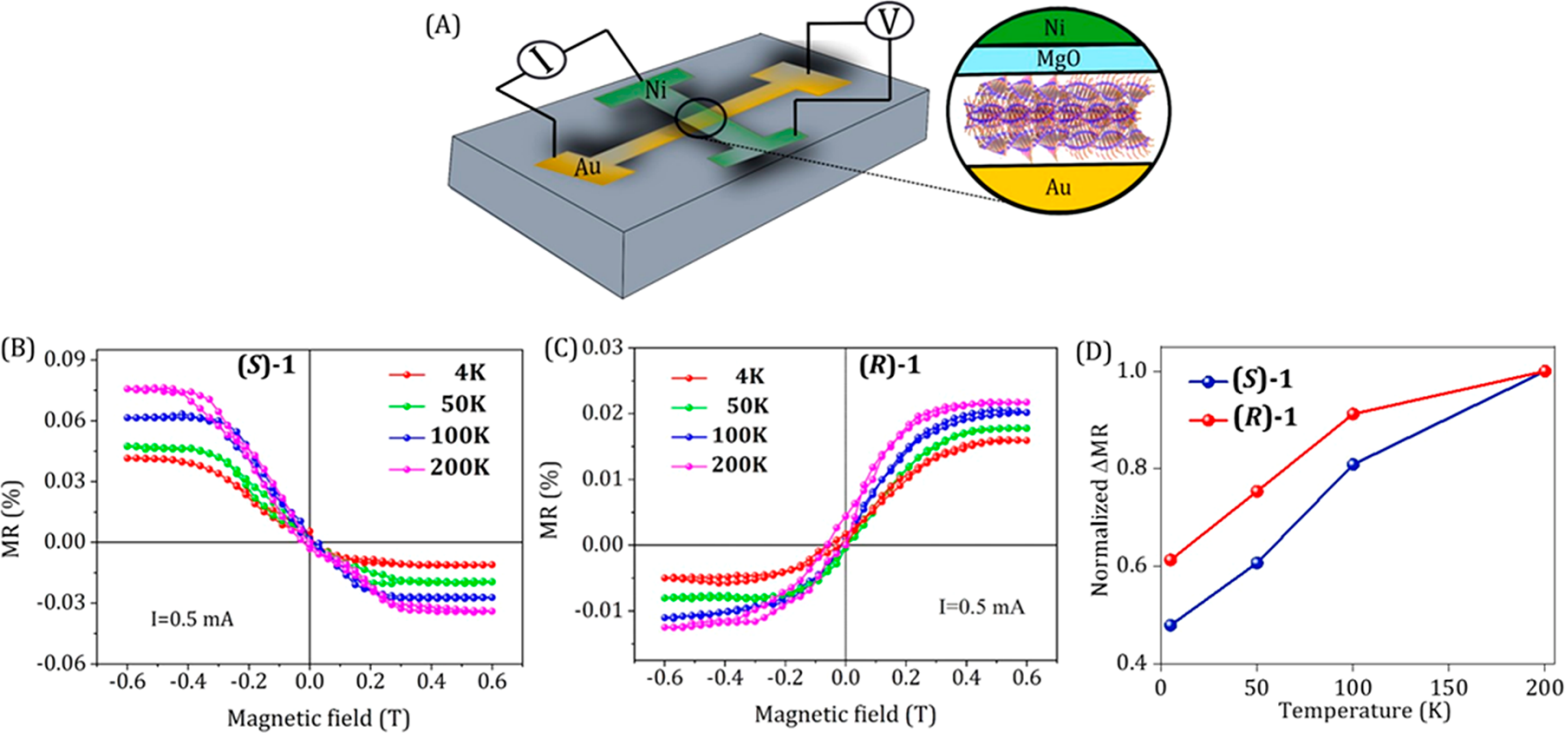
Temperature-dependent magnetoresistance studies. (A) Schematic representation of the four-probe magnetoresistance (MR) measurement setup with bottom gold and top Ni electrode. Panels B and C present the magnetoresistance curves for films obtained from **(*****S*****)-1** and **(*****R*****)-1** respectively, as a function of magnetic field between −0.6 and 0.6 T at different temperatures. The measurements were performed at a constant current of 0.5 mA. Panel D represents the normalized ΔMR values as a function of temperature, where, ΔMR (%) = |MR (%)|_–0.6T_ + |MR (%)|_+0.6T_.

To obtain insight into the temperature dependence of spin transport in the fibers, we applied a spin-valve configuration in which the magnetoresistance (MR) of the fibers is measured. Figure 5A presents a scheme of the device structure. The crossbar geometry measures the resistance of the device by the standard 4-probe configuration, when the magnetoresistance is defined as MR (%) = , where *R*_H=0_ and *R*_H_ are the zero-field and infield resistances, respectively.^35^Figure 5B, C show the magnetoresistance curves for films formed using **(*****S*****)-1** and **(*****R*****)-1**, respectively, as a function of the magnetic field between −0.6 and 0.6 T at different temperatures. The measurements were performed at a constant current of 0.5 mA. The low MR values obtained are a result of the fibers being deposited on the surface randomly, so that the layer had a large number of pin holes through which current is flowing directly without passing through the chiral fibers. This large “background current” is the reason for the low values of MR. However, despite the signal being small, the signal-to-noise ratio is excellent and the reproducibility is high. Like in former studies with chiral molecules, an asymmetric curve of the magnetoresistance as a function of magnetic field is observed.^35^ We found a correlation between the mc-AFM results and the MR studies. For instance, in the case of **(*****R*****)-1**, using the mc-AFM, when the magnet is in DOWN configuration, the current has a higher value. Consistently in the MR studies, the resistance is lower for a negative magnetic field which corresponds to field DOWN. MR devices constructed from achiral fibers when assembled in an achiral solvent showed a symmetric nature of MR curves (SI, Figure S6), confirming that the chirality of the systems leads to asymmetric MR curves.

The possible role of phonons in the CISS effect was discussed recently.^39,40^ It was proposed that phonons and polarons can enhance the spin polarization. From Figure 5B,C the temperature-dependent MR signals at magnetic fields of ±0.6 T were extracted and are plotted in Figure 5D after normalization. The spin polarization drops by about 50% by cooling the system from 200 to 4 K. The results point to the possibility that indeed phonons enhance the spin polarization, as suggested theoretically.

## Conclusions

In this work we successfully demonstrated that the CISS effect can occur in supramolecular polymers exclusively consisting of achiral monomers, when asymmetry results from an external, removable source such as chiral solvents. The spin selectivity is shown to be related to the excess of preferred handedness of the supramolecular polymers. It was also shown that the degree of spin polarization observed for electrons transferred through the fibers depends on the layer thickness of the stacks. From the thickness-dependent studies, it is possible to infer that at large thicknesses there are two spin related mechanisms active. The first is the CISS effect that polarizes the spin, while the second relates to scattering of the electrons, reducing the spin polarization. Temperature-dependent studies provide the first support for theoretical work that suggested that phonons may enhance the spin polarization. These insights clarify our understanding of the CISS effect being a result of chiral superstructures rather than only chiral molecules. These conclusions open new pathways for the construction of novel organic spintronic devices. The work further provided new insights into the mechanism behind the CISS based phenomena.

## Supporting Information Available

Experimental details, synthesis, spectroscopic characterization, magnetic conductive probe atomic force microscopy, magnetoresistance measurement (PDF)
