# Supplementary material for: Spin Filtering in Supramolecular Polymers Assembled from Achiral Monomers Mediated by Chiral Solvents
Source: J Am Chem Soc. 2021 Apr 30;143(18):7189–95. doi: 10.1021/jacs.1c02983 (PMC8297732; doi:10.1021/jacs.1c02983)
Supplement: Supplementary file 1 — ja1c02983_si_001.pdf [file ja1c02983_si_001.pdf]

Supporting Information

Spin Filtering in Supramolecular Polymers Assembled from Achiral Monomers Mediated by Chiral Solvents

Amit Kumar Mondal[1†], Marco D. Preuss[2†], Marcin L. Ślęczkowski[2], Tapan Kumar Das[1], Ghislaine Vantomme[2], E.W. Meijer[2]*, and Ron Naaman[1]*

[1]Department of Chemical and Biological Physics, Weizmann Institute of Science, Rehovot 76100, Israel.

[2]Institute for Complex Molecular Systems and Laboratory of Macromolecular and Organic Chemistry, Eindhoven University of Technology, P.O. Box 513, 5600 MB Eindhoven, The Netherlands.

Email: ron.naaman@weizmann.ac.il, e.w.meijer@tue.nl

Contents

1. Materials and Methods 3
   1.1. General Considerations 3
   1.2. Characterization Methods 3
   1.3. Sample Preparation Protocol 3
2. Synthetic Procedures 4
   2.1. Synthesis of Triphenylene-2,6,10-tricarboxamides (5) 4
      2,6,10-trimethyl-1,2,3,4,5,6,7,8,9,10,11,12-dodecahydrotriphenylene (2) 5
      2,6,10-Trimethyltriphenylene (3) 5
      Triphenylene-2,6,10-tricarboxylic acid (4) 5
      General Procedure for the Synthesis of Triphenylene-2,6,10-tricarboxamides (5) 6
      N^2,N^6,N^{10}-Tris(octyl)triphenylene-2,6,10-tricarboxamide (5-(*n*-*8*)) 6
      N^2,N^6,N^{10}-Tris(decyl)triphenylene-2,6,10-tricarboxamide (5-(*n*-*10*)) 6
   2.2. Synthesis of optically active solvents 7
      (*S*)-3,7-dimethyloctan-1-ol ((*S*)-7) 7
      (*S*)-1-Cl-3,7-dimethyloctane ((*S*)-8) 7
      (*R*)-3,7-dimethyloctan-1-ol ((*R*)-7) 8
      (*R*)-1-Cl-3,7-dimethyloctane ((*R*)-8) 8
      (*S*)-1-chloro-2-methylbutane ((*S*)-10) 8
3. Magnetic Conductive Probe Atomic Force Microscopy (mc-AFM) 10
      Magnetic conductive probe atomic force microscopy (mc-AFM) sample preparation: 10
      CISS effect measurement using mc-AFM: 10
      Device Fabrication for magnetoresistance measurement: 10
4. Spectroscopic characterization 15
   4.1. Solution Based Spectroscopic Characterization 15
      UV spectra of 1 and 2 in (*S*)-CldMeOct and decaline (achiral) 15
      Full CD spectra for 1 in (*S*)-ClMeBu of different enantiomeric excess (*ee*) 16
      CD-spectra of the pure solvents 16
   4.2. Solid state UV and CD characterization 17
5. Spectroscopic Characterization of Final Compounds 20

1. Materials and Methods

1.1. General Considerations

All used materials were purchased from commercial sources and were used without further purification unless otherwise noted. (*S*)- and (*R*)-citronellol were purchased from Sigma-Aldrich. The optical purity was elucidated prior to use. For (*R*)-citronellol ($[\alpha]_D^{20}$ = -4.9°) a lower optical purity was observed than for the naturally occurring (*S*)-citronellol ($[\alpha]_D^{20}$ = +5.3°). Racemic 1-chlor-2-methylbutane was purchased from Sigma-Aldrich and distilled prior to use. Air and moisture sensitive reactions were carried out under argon atmosphere using SCHLENK techniques. Solids and liquids were added in argon counterflow. Glassware was heat gun dried under high vacuum prior to use. Solvents used for air or moisture sensitive reactions were either purchased anhydrous or taken from MBraun solvent purification system. Thin layer chromatography (TLC) was carried out using silica coated aluminum sheets (60-F254 Merck) and visualized by UV light at 254 nm and 366 nm.

1.2. Characterization Methods

NMR analysis was carried out using Bruker Mercury Vx 400 MHz spectrometers using deuterated solvents purchased from Cambridge Isotope Laboratories. Chemical shifts (d) are reported in parts-per-million (ppm) relative to the residual solvent protons (^{1}H-NMR) or the deuterium coupled 13C solvent signal (^{13}C-NMR). Coupling constants of protons (J) are reported in Hertz (Hz). Spin multiplicities are reported using the following abbreviations or appropriate combinations of such: s (singlet), d (doublet), t (triplet), m (multiplet). The NMR spectra were processed using MestReNova x64 14.01. (Mestrelab Research S.L.).

Mass spectrometry analysis was performed using matrix assisted laser desorption/ionization time-of-flight (MALDI-TOF) on a Bruker AutoFlex spectrometer using a-cyano-4-hydroxycinnamic acid (CHCA) as matrix.

Fourier-transform infrared spectra were recorded on a Perkin Elmer FT-IR Spectrum Two apparatus and reported in wavenumbers (u).

CD measurements were performed using JASCO J-815 CD spectrometer equipped with a JASCO Peltier PFD-425S/15 with a temperature range of 263 K to 383 K using the following settings; sensitivity: Standard, D.I.T: 0.25 s, bandwidth: 1 nm, scanning speed: 50 nm/min, data pitch: 0.2 nm. Variable temperature CD (VT-CD) experiments were performed using the following settings; sensitivity: Standard, D.I.T: 2 s, bandwidth: 1 nm, cooling rate: 0.5 °C/min, data pitch: 0.1 °C. All spectroscopic measurements were performed using HELLMA quartz cuvettes with an optical pathlength of 1 mm and 9 mm metal spacer as heat bridge. Bulk measurements were performed on 2 cm x 2 cm x 0.1 cm (l x w x t) quartz slides. Solid state measurements were performed using a solid-state holder with a circular opening of 12 mm^2 (d=4 mm).

Optical rotation was measured using a JASCO DIP-370 Digital Polarimeter at room temperature. Samples were placed in a quartz cuvette with an optical pathlength of 50 mm.

1.3. Sample Preparation Protocol

Solvent addition was performed using Gilson MICROMAN Positive-Displacement Pipets (range: 3 mL - 25 mL, 25 mL – 100 mL, 50 mL – 250 mL and 100 mL – 1000 mL). Compounds were weighted using a Sartorius Lab Instrument microbalance (d=0,0001 mg). From each compound a 75 μM stock solution was prepared according to the following procedure: The

desired compound was weighed in on an aluminum weighting boat, placed in a 4 mL screw-cap vial and mixed with the according amount of solvent. The samples were placed in a sand bath at 120 °C and stirred using a magnetic stirring bar. After all compound had dissolved, the samples were taken out of the heating bath and cooled to room temperature by leaving them on the bench. For preparation of mixtures in 1-chloro-2-methylbutane of different enantiomeric excess, two 75 μM stock solutions in racemic and optically pure solvent were prepared as described before. Equilibration of the samples was performed at 70 °C. To obtain solutions of different *ee*, the stock solutions were mixed in the appropriate ratio. After mixing, the samples were equilibrated at 70 °C before measuring CD.

For each sample the absorption profile at room temperature was collected to ensure matching concentrations between the samples. If necessary, concentration adjustments were carried out by adding more solvent.

CD bulk measurements were performed by static spin-coating on quartz glass cleaned by subsequent sonication for 15 minutes in acetone, DI water and isopropanol. 5 μL of the desired 75 μM solution was deposited in the center of the substrate and left for 60s before starting to spin at 300 rpm for 60s. This process was repeated four times, till a total volume of 20 μL was deposited.

It is important to notice that solvent purity is of high importance for obtaining reproducible results. All solvents were vacuum distilled prior to use and stored over molecular sieves (3 Å) and inert atmosphere to minimize possible moisture effects.

2. Synthetic Procedures

2.1. Synthesis of Triphenylene-2,6,10-tricarboxamides (5)

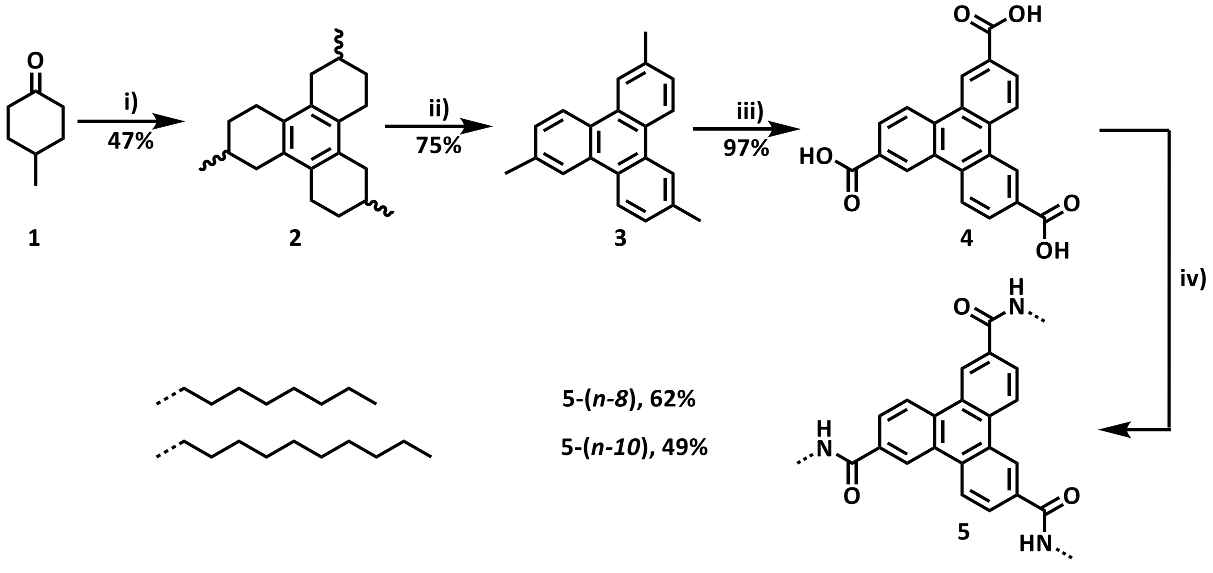

Scheme 1 Synthetic overview of triphenylene-2,6,20-tricarboxamide derivates **5**. Reagents and conditions: (i) $ZrCl_4$, 170 °C; (ii) 10 wt% Pd/C, triglyme, 220 °C; (iii) $Na_2Cr_2O_7$, H_2O, 250 °C; (iv) aliphatic amine, TBTU, DMAP, DIPEA, DMF, 70 °C.

Experimental procedures for the Synthesis of triphenylene-2,6,10-tricarboxylic acid was derived from procedures published by Bock *et al.*[1] and Shirai *et al.*[2] starting from 4-

methylcyclohexanone. Amidation reactions were carried out following the general protocol recently published by the Meijer-group.[3]

2,6,10-trimethyl-1,2,3,4,5,6,7,8,9,10,11,12-dodecahydrotriphenylene (2)
4-Methylcyclohexanone (50 g, 450 mmol) (**1**) was placed in a round-bottom flask equipped with a magnetic stirrer and a reflux condenser. Then ZrCl4 (4 g, 20 mmol) was added and the mixture was stirred at 170 °C for 5 hours. The progress was monitored by TLC (heptane / EtOAc 9:1). On completion the reaction mixture was cooled down to room temperature. The resulting solid was re-dissolved in hot $CHCl_3$ (400 mL) and an insoluble residue was filtered off at elevated temperature. The filtrate was concentrated under reduced pressure to yield 41 g of an orange solid. The product was recrystallized from n-butanol to yield 19.2 g of white crystals. The mother liquor was collected and concentrated under reduced pressure to give 18 g of a brown solid, which was divided into 2 parts of 9 grams and subjected to flash column chromatography using eluent gradient from heptane to a 9:1 heptane/EtOAc mixture. The resulting product was recrystallized from n-butanol and combined with the previously recrystallized fraction to give the title compound as colorless crystals (19.7 g, 69.73 mmol, 47%). ^{1}H-NMR (400 MHz, $CDCl_3$): δ (ppm) = 2.82 (m, 3H); 2.74-2.43 (m, 6H); 2.22 – 2.01 (m, 3H); 1.93 (m, 3H); 1.77 (m, 3H); 1.35 (m, 3H); 1.11 (m, 9H). ^{13}C-NMR (100 MHz, $CDCl_3$): δ = sym: 132.52; 132.12; 36.16; 31.54; 29.21; 27.55; 22.66; dissym: 132.56; 132.30; 132.24; 132.23; 132.05; 132.02; 35.98; 35.50; 35.24; 31.41; 31.37; 30.94; 29.09; 28.94; 28;56; 27.28; 26.68; 26.07; 22.45; 22.41; 21.84. FT-IR (cm^{-1}) = 2982-2772 (υ-C-H, s); 1454 (δ-C-H, m). **MS (MALDI-TOF):** *m/z* calcd. For $C_{21}H_{30}^{rad+}$ [M^{rad+}] = 282.23; found: 282.26.

2,6,10-Trimethyltriphenylene (3)
In a 50 mL round-bottom flask equipped with stirring bar and reflux condenser, 3.5 g of 2,6,10-trimethyl-1,2,3,4,5,6,7,8,9,10,11,12-dodecahydrotriphenylene (12.39 mmol, 1.0 eq) and 35 mL triglyme were bubbled with argon for 15 min before adding 0.4 g Pd/C (10 wt%). The resulting mixture was heated to 220 °C overnight. The progression of the reaction was monitored by TLC (9:1 heptane/ ethyl acetate). Upon completion, the mixture was cooled to room temperature, diluted with 100 mL chloroform and heated to reflux. The hot solution was filtered through a pad of Celite, and the latter was washed with hot chloroform till no more product could be observed in the filtrate. The combined organic layers were concentrated under reduced pressure to give a pale-yellow liquid which crystallized in the freezer over the weekend. Remaining liquid was filtered off to give the product as a pale yellow solid (2.5 g, 9.29 mmol, 75%). **^{1}H-NMR, COSY (400 MHz, Chloroform-*d*)** δ/ppm = 8.52 (d, J = 8.4 Hz, 3H), 8.40 (s, 3H), 7.44 (dd, J = 8.3, 1.8 Hz, 3H), 2.61 (s, 9H). **^{13}C-NMR, HSQC, HMBC (101 MHz, Chloroform-*d*)**: δ/ppm = 136.75, 130.13, 128.25, 127.17, 123.29, 123.22, 21.97. **FT-IR** (ATR): ν (cm^{-1}) = 2913, 1614, 1500, 1406, 1038, 870, 813, 763, 591. **MS (MALDI-TOF):** *m/z* calcd. For $C_{21}H_{18}^{rad+}$ [M^{rad+}] = 270.14; found: 270.24.

Triphenylene-2,6,10-tricarboxylic acid (4)
In a 15 mL steel reactor equipped with stirring bar, 0.3 g 2,6,10-trimethyltriphenyl (1.11 mmol, 1.0 eq.) and 1.6 g $Na_2Cr_2O_7$ (5.37 mmol, 4.8 eq.) were dispersed in 4 mL water. The suspension was pre-stirred to ensure well mixing before closing the reactor and heating to 250 °C over the weekend. The mixture was cooled to room temperature and diluted with 5 mL water. The green suspension was filtered to give a yellow filtrate. The latter was acidified with concentrated HCl and the resulting precipitate was filtered off, washed with water and dried in the vacuum oven at 90 °C overnight. The product was isolated as yellow solid (388 mg, 1.08 mmol, 97%). **^{1}H-**

NMR, COSY (400 MHz, DMSO-*d6*) δ/ppm = 13.35 (s, 3H), 9.27 (s, 3H), 8.88 (d, *J* = 8.8 Hz, 3H), 8.26 (dd, *J* = 8.5, 1.6 Hz, 3H). **^{13}C-NMR (101 MHz, DMSO-*d6*)**: δ/ppm = 167.07, 132.63, 130.19, 128.81, 128.29, 125.49, 124.28. **FT-IR** (ATR): ν (cm^{-1}) = 2996, 1689, 1615, 1433, 1276, 846, 717, 488.

General Procedure for the Synthesis of Triphenylene-2,6,10-tricarboxamides (5)
In a 25 mL two-necked round-bottom flask equipped with stirring bar and argon-inlet, 50 mg triphenylene-2,6,10-tricarboxylic acid (0.14 mmol, 1.0 eq.) were dissolved at 70 °C in 0.5 mL dry DMF. After addition of 148 mg TBTU (0.46 mmol, 3.3 eq.) in 0.5 mL dry DMF, 145 μL DIPEA (0.83 mmol, 6 eq.) and the required amine (0.46 mmol, 3.3 eq.) were subsequently added. The solution was allowed to stir overnight at 70 °C. After cooling to room temperature, the solution was diluted with 200 mL dichloromethane and the organic layer was subsequently washed with 50 mL water, 50 mL 1 M HCl (aq.), 50 mL 1 M NaOH (aq.), 50 mL water and 50 mL brine. The organic phase was dried over magnesium sulfate, filtered and concentrated under reduced pressure. The crude product was purified by recrystallization from methanol and filtered using a G4 frit.

***N^2,N^6,N^{10}*-Tris(octyl)triphenylene-2,6,10-tricarboxamide (5-(*n-8*))**
The title compound was isolated by recrystallization from methanol as colorless solid (60 mg, 0.09 mmol, 62%). **^{1}H-NMR (400 MHz, $CDCl_3$ + TFA-*d*)**** δ/ppm = 10.46 (s, 3H), 8.29 (s, 3H, *H*-1, *H*-5, *H*-9), 7.91 (d, J = 8.5 Hz, 3H, *H*-4, *H*-8, *H*-12), 7.60 (d, J = 8.5 Hz, 3H, *H*-3, *H*-7, *H*-11), 3.57 (t, J = 7.5 Hz, 6H, CH_2-1, CH_2-1’, CH_2-1’’), 1.76 (p, J = 7.3 Hz, 6H, CH_2-2, CH_2-2’, CH_2-2’’), 1.60 – 1.24 (m, 30H, CH_2-3, CH_2-3’, CH_2-3’’, CH_2-4, CH_2-4’, CH_2-4’’, CH_2-5, CH_2-5’, CH_2-5’’, CH_2-6, CH_2-6’, CH_2-6’’, CH_2-7, CH_2-7’, CH_2-7’’), 0.91 (t, J = 6.4 Hz, 9H, *C*H_3-8, *C*H_3-8’, *C*H_3-8’’). **^{13}C-NMR (100 MHz, $CDCl_3$ + TFA-*d*)****: δ/ppm = 169.36 (*C*ONH), 131.96 (*C*$_q$-4a, *C*$_q$-8a, *C*$_q$-12a), 131.13 (*C*$_q$-4b, *C*$_q$-8b, *C*$_q$-12b), 128.18 (*C*$_q$-2, *C*$_q$-6, *C*$_q$-10), 125.55 (*C*-3, *C*-7, *C*-11), 123.62 (*C*-4, *C*-8, *C*-12), 123.31 (*C*-1, *C*-5, *C*-9), 41.50 (*C*H_2-1, *C*H_2-1’, *C*H_2-1’’), 31.94 (*C*H_2-7, *C*H_2-7’, *C*H_2-7’’), 29.38 (*C*H_2-4, *C*H_2-4’, *C*H_2-4’’), 29.35 (*C*H_2-5, *C*H_2-5’, *C*H_2-5’’), 29.22 (*C*H_2-2, *C*H_2-2’, *C*H_2-2’’), 27.16 (*C*H_2-3, *C*H_2-3’, *C*H_2-3’’), 22.78 (*C*H_2-6, *C*H_2-6’, *C*H_2-6’’), 14.19 (*C*H_3-8, *C*H_3-8’, *C*H_3-8’’). **FT-IR** (ATR): ν (cm^{-1}) = 3244 (υ_{N-H}, m); 1632 (amide I, s); 1547 (amide II, s). **MS (MALDI-TOF)**: *m/z* calcd. for $C_{45}H_{63}N_3O_3^+$ [M+H$^+$] = 694.49; found: 694.49.

***N^2,N^6,N^{10}*-Tris(decyl)triphenylene-2,6,10-tricarboxamide (5-(*n-10*))**
The title compound was isolated by recrystallization from methanol as colorless solid (54 mg, 0.07 mmol, 49%). **^{1}H-NMR, COSY (400 MHz, $CDCl_3$ + TFA-*d*)**** δ/ppm = 8.46 (s, 3H, *H*-1, *H*-5, *H*-9), 8.09 (d, *J* = 8.6 Hz, 3H, *H*-4, *H*-8, *H*-12), 7.67 (d, *J* = 8.4 Hz, 3H, *H*-3, *H*-7, *H*-11), 3.60 (t, *J* = 7.5 Hz, 6H, CH_2-1, CH_2-1’, CH_2-1’’), 1.76 (p, *J* = 7.4 Hz, 6H, CH_2-2, CH_2-2’, CH_2-2’’), 1.51 – 1.19 (m, 48H, CH_2-3, CH_2-3’, CH_2-3’’, CH_2-4, CH_2-4’, CH_2-4’’, CH_2-5, CH_2-5’, CH_2-5’’, CH_2-6, CH_2-6’, CH_2-6’’, CH_2-7, CH_2-7’, CH_2-7’’, CH_2-8, CH_2-8’, CH_2-8’’, CH_2-9, CH_2-9’, CH_2-9’’), 0.89 (t, *J* = 6.5 Hz, 9H, CH_3-10, CH_3-10’, CH_3-10’’). **^{13}C-NMR, HSQC, HMBC (101 MHz, $CDCl_3$ + TFA-*d*)****: δ/ppm = 170.03 (*C*ONH), 132.36 (*C*$_q$-4a, *C*$_q$-8a, *C*$_q$-12a), 131.01 (*C*$_q$-4b, *C*$_q$-8b, *C*$_q$-12b), 128.47 (*C*$_q$-2, *C*$_q$-6, *C*$_q$-10), 125.75 (*C*-3, *C*-7, *C*-11), 123.93 (*C*-4, *C*-8, *C*-12), 123.65 (*C*-1, *C*-5, *C*-9), 41.80 (*C*H_2-1, *C*H_2-1’, *C*H_2-1’’), 32.05 (*C*H_2-8, *C*H_2-8’, *C*H_2-8’’), 29.71*, 29.69*, 29.47*, 29.42*, 29.16 (*C*H_2-2, *C*H_2-2’, *C*H_2-2’’), 27.13 (*C*H_2-3, *C*H_2-3’, *C*H_2-3’’), 22.83 (*C*H_2-9, *C*H_2-9’, *C*H_2-9’’), 14.17 (*C*H_3-10, *C*H_3-10’, *C*H_3-10’’). **FT-IR** (ATR): ν (cm^{-1}) = 3242 (υ_{N-H}, m), 2922, 2853, 1633 (amide-I, s), 1549 (amide-II,

s), 1314, 1283, 740, 704. **MS (MALDI-TOF)**: *m/z* calcd. for $C_{51}H_{76}N_3O_3^+$ [M+H$^+$] = 778.59; found: 778.60.

*(CH$_2$-4, CH$_2$-4', CH$_2$-4'', CH$_2$-5, CH$_2$-5', CH$_2$-5'', CH$_2$-6, CH$_2$-6', CH$_2$-6'', CH$_2$-7, CH$_2$-7', CH$_2$-7'') no clear assignment possible

**Addition of TFA-*d* was necessary to break the aggregates and make NMR characterization possible.

2.2. Synthesis of optically active solvents

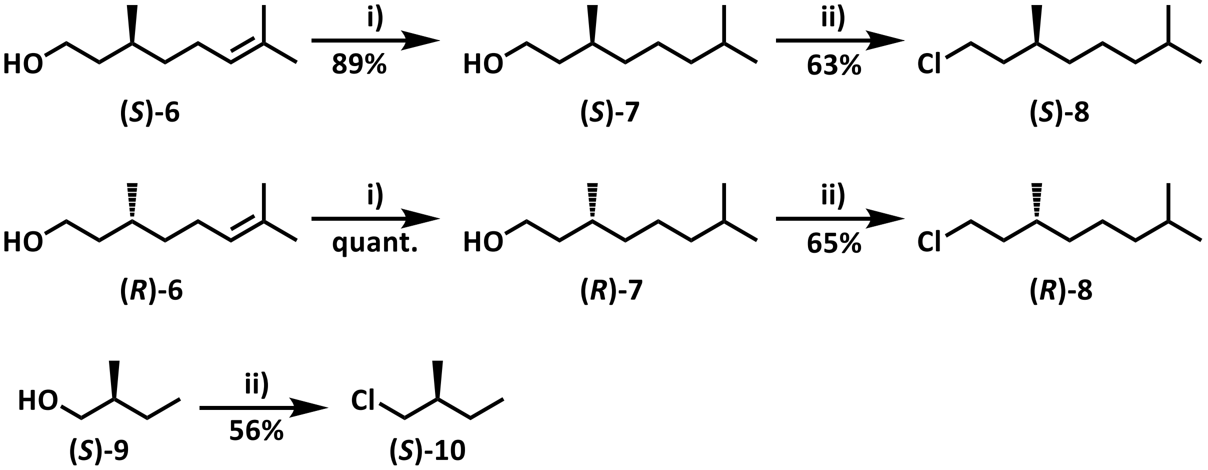

Scheme 2 Synthetic overview of optical active solvents **(*S*)-8**, **(*R*)-8** and **(*S*)-10**. Reagents and conditions: (i) H_2, 10 wt% Pd/C, EtOAc, 24h, rt; (ii) $SOCl_2$, Pyridine, overnight, 60 °C.

The synthesis of optically active solvents was slightly modified from previously published synthetic procedures.[3]

(*S*)-3,7-dimethyloctan-1-ol ((*S*)-7)

The procedure was adapted as previously published. A 250 mL Parr reaction vessel was charged with 57.9 g (370.8 mmol, 1 eq.) (*S*)-3,7-dimethyl-oct-6-en-1-ol and diluted with 40 mL ethyl acetate. The mixture was bubbled with nitrogen for 15 min and 0.5 g 10 wt% activated Pd/C was added. The reaction vessel was installed in a Parr apparatus, placed under hydrogen and shaken until no further pressure reduction was observed. The progress of hydrogenation was additionally monitored using ^{1}H-NMR. For reaching full conversion, addition of extra 50 mg of 10 wt% Pd/C was necessary over the course of reaction. The reaction mixture was diluted with 20 mL ethyl acetate and filtered through Celite. The filter cake was further flushed with 100 mL of ethyl acetate. The combined organic layers were concentrated under reduced pressure and dried under high vacuum. The product was isolated as a colorless liquid (56.8 g, 359.2 mmol, 96%). **^{1}H-NMR (400 MHz, CDCl$_3$)**: δ (ppm) = 3.73 – 3.59 (m, 2H), 1.66 – 1.05 (m, 10H), 0.98 – 0.73 (m, 9H). **^{13}C-NMR (101 MHz, CDCl$_3$)**: δ (ppm) = 61.37, 40.13, 39.39, 37.51, 29.64, 28.10, 24.81, 22.83, 22.72, 19.77. **FT-IR** (ATR): ν (cm^{-1}) = 3330 (br), 2955, 2927, 1464, 1382, 1055.

(*S*)-1-chloro-3,7-dimethyloctane ((*S*)-8)

In a 500 mL three necked round bottom flask equipped with a mechanical stirrer and dropping funnel, 43.6 mg (275.6 mmol, 1.0 eq.) (*S*)-3,7-dimethyloctan-1-ol and 22.2 mL freshly distilled pyridine (275.6 mmol, 1.0 eq.) were heated to 60 °C. 30 mL thionyl chloride (413.4 mmol, 1.5 eq.) were added dropwise (1 drop per second) and the reaction was allowed to stir overnight

at 60 °C. The reaction mixture was cooled to room temperature, allowing the pyridinium chloride to crystallize. The remaining liquid phase was transferred into a separatory funnel and extracted with 100 mL water, 100 mL saturated $NaHCO_3$ (strong foaming) and 100 mL brine respectively. The crude product was dried over magnesium sulfate and purified by fractioned vacuum distillation (bp = 38 °C, 1.1 mbar) over activated charcoal using a Vigreux column. In order to obtain solvent in spectroscopic quality, the distillate was further stirred overnight with a mixture of silicagel, neutral aluminum oxide, potassium carbonate and magnesium sulfate (ca 0.5 g each). After filtration, the product was isolated as a colorless liquid (30.4 g, 172.7 mmol, 63%).**^{1}H-NMR (400 MHz, $CDCl_3$)**: δ (ppm) = 3.66 – 3.48 (m, 2H), 1.87 – 1.72 (m, 1H), 1.70 – 1.44 (m, 3H), 1.39 – 1.20 (m, 3H), 1.20 – 1.05 (m, 3H), 1.05 – 0.66 (m, 9H). **^{13}C-NMR (101 MHz, $CDCl_3$)**: δ (ppm) = 43.53, 39.96, 39.33, 36.99, 30.55, 28.10, 24.71, 22.83, 22.73, 19.23. **FT-IR** (ATR): ν (cm^{-1}) = 2956, 2927, 1464, 1383, 1367, 727, 659. **$[\alpha]_D^{20}$** = +2.7°.

(*R*)-3,7-dimethyloctan-1-ol ((*R*)-7)
The procedure was adapted as previously published. A 250 mL Parr reaction vessel was charged with 30.0 g (191.2 mmol, 1 eq.) (*R*)-3,7-dimethyl-oct-6-en-1-ol and diluted with 40 mL ethyl acetate. The mixture was bubbled with nitrogen for 15 min and 0.3 g 10 wt% activated Pd/C was added. The reaction vessel was installed in a Parr apparatus, placed under hydrogen and shaken until no further pressure reduction was observed. The progress of hydrogenation was additionally monitored using ^{1}H-NMR. For reaching full conversion, addition of extra 50 mg of 10 wt% Pd/C was necessary over the course of reaction. The reaction mixture was diluted with 20 mL ethyl acetate and filtered through Celite. The filter cake was further flushed with 100 mL of ethyl acetate. The combined organic layers were concentrated under reduced pressure and dried under high vacuum. The product was isolated as a colorless liquid (30.2 g, 190.9 mmol, 99%). **^{1}H-NMR (400 MHz, $CDCl_3$)**: δ (ppm) = 3.76 – 3.61 (m, 2H), 1.66 – 1.47 (m, 4H), 1.34 – 1.08 (m, 6H), 0.92 – 0.83 (m, 9H).

(*R*)-1-chloro-3,7-dimethyloctane ((*R*)-8)
In a 500 mL three necked round bottom flask equipped with a mechanical stirrer and dropping funnel, 30.2 g (190.9 mmol, 1.0 eq.) (*R*)-3,7-dimethyloctan-1-ol and 15.4 mL freshly distilled pyridine (190.9 mmol, 1.0 eq.) were heated to 60 °C. 20.4 mL thionyl chloride (286.3 mmol, 1.5 eq.) were added dropwise (1 drop per second) and the reaction was allowed to stir overnight at 60 °C. The reaction mixture was cooled to room temperature, allowing the pyridinium chloride to crystallize. The remaining liquid phase was transferred into a separatory funnel and extracted with 100 mL water, 100 mL saturated $NaHCO_3$ (strong foaming) and 100 mL brine respectively. The crude product was dried over magnesium sulfate and purified by fractioned vacuum distillation (bp = 38 °C, 1.1 mbar) over activated charcoal using a Vigreux column. In order to obtain solvent in spectroscopic quality, the distillate was further stirred overnight with a mixture of silicagel, neutral aluminum oxide, potassium carbonate and magnesium sulfate (ca 0.5 g each). After filtration, the product was isolated as a colorless liquid (21.9 g, 123.69 mmol, 65%).**^{1}H-NMR (400 MHz, $CDCl_3$)**: δ (ppm) = 3.64 – 3.48 (m, 2H), 1.86 – 1.73 (m, 1H), 1.72 – 1.44 (m, 3H), 1.38 – 1.20 (m, 3H), 1.20 – 1.05 (m, 3H), 0.97 – 0.82 (m, 9H). **^{13}C-NMR (101 MHz, $CDCl_3$)**: δ (ppm) = 43.54, 39.96, 39.33, 36.99, 30.55, 28.10, 24.71, 22.83, 22.74, 19.23. **FT-IR** (ATR): ν (cm^{-1}) = 2956, 2927, 1464, 1383, 1367, 727, 659. **$[\alpha]_D^{20}$** = -2.5°.

(*S*)-1-chloro-2-methylbutane ((*S*)-10)
A method from previously published report was adapted. (*S*)-2-methylbutan-1-ol (247 mL, 2.27 mol) and freshly distilled pyridine (183 mL, 2.27 mol) were placed in a 1 L 3-neck round-

bottomed flask equipped with a mechanical stirrer and dropping funnel. The mixture was heated to 60 °C and subsequently, freshly distilled thionyl chloride (246 mL, 3.40 mol) was added dropwise over 4 hours. Upon addition of ca. 1 eq. of $SOCl_2$, the reaction mixture became a slurry due to the crystallization of pyridinium chloride. Upon subsequent addition of the remaining $SOCl_2$, a brown biphasic mixture was obtained. The progress of the reaction was monitored by ^{1}H-NMR. The reaction was regarded as complete, when a characteristic quartet of doublets at 3.47 ppm was the only group of signals between 5 ppm and 3 ppm and signals from other intermediates corresponding to possible sulfite that formed over the course of the reaction were not present anymore. On completion, the biphasic mixture was transferred to a separatory funnel without cooling and quickly separated while the bottom, inorganic phase had not crystallized yet. The organic upper layer was subsequently washed with water, saturated aqueous $NaHCO_3$, water and brine. During the washing stages, the formation of an emulsion is possible. After washing, the combined organic fractions were dried over magnesium sulfate and after filtering off the drying agent, 150 mL of a brown liquid was obtained. In order to yield the solvent in suitable quality for spectroscopic measurements, three fractional distillations under reduced pressure (p = 190 mbar, boiling point = 60 °C) were carried out: After the first distillation, the distillate was collected and redistilled twice from activated charcoal. Finally, the liquid was stirred overnight with a mixture containing activated charcoal, silica gel, neutral alumina and potassium carbonate (ca. 1 gram each) and subsequently filtered off to yield 135 mL (56%) of (*S*)-ClMeBu, which was stored over 3Å molecular sieves. **^{1}H NMR (400 MHz, $CDCl_3$):** δ (ppm) = 3.52 – 3.37 (m, 2H), 1.81 – 1.67 (m, J = 6.2 Hz, 1H), 1.58 – 1.43 (m, 1H), 1.34 – 1.18 (m, 1H), 1.00 (d, J = 6.7 Hz, 3H), 0.91 (t, J = 7.4 Hz, 3H). ^{13}C NMR (101 MHz, $CDCl_3$): δ (ppm) = 51.02, 37.24, 26.81, 17.50, 11.34. **FT-IR** (ATR): ν (cm^{-1}) = 2965, 1459, 1380, 726, 681.

3. Magnetic Conductive Probe Atomic Force Microscopy (mc-AFM)

Magnetic conductive probe atomic force microscopy (mc-AFM) sample preparation:
Substrate surfaces were prepared by sputtering a 120 nm layer of nickel, followed by a 10 nm layer of gold on top of a silicon wafer with a 2 μm thermal silicon oxide layer, with a 10 nm titanium layer for adhesion. The Ni/Au surfaces was used to enable magnetic field-induced spin-polarization of the electrons injected from the surface to the nanofibers. All surfaces were cleaned by first immersing in boiling acetone and then in ethanol for 10 min, followed by a UV-ozone cleaning for 15 min and a final incubation in warm ethanol for 40 min. Samples for magnetic conductive probe atomic force microscopy (mc-AFM) measurements were prepared by drop-casting the solutions of nanofibers on the surface.

CISS effect measurement using mc-AFM:
Magnetic field-dependent current vs. voltage (*I-V*) characteristics of the nanofibers were obtained using a multimode magnetic scanning probe microscopy (SPM) system built with Beetle Ambient AFM and an electromagnet equipped with R9 electronics controller (RHK Technology). Voltage spectroscopy for *I-V* measurements were performed by applying voltage ramps with a Pt tip (DPE-XSC11, μmasch with spring constant 3-5 Nm^{-1}) in contact with the sample at an applied force of 4-6 nN. At least 40-50 *I-V* curves were scanned in an applied magnetic field of 0.50 T for both magnetic field orientation (field UP and DOWN).

Device Fabrication for magnetoresistance measurement:
The device for the magnetoresistance measurement was fabricated as follows. The device has a vertical structure. The first layer is Au with Ti as adhesion layer on top of the Si/SiO_2 substrate (111). The Au and Ti lines, 4 μm width and 80 nm and 8 nm thick correspondingly, were fabricated by optical lithography. The solution of nanofibers was drop cast on the gold line. Layer of 2.5 nm thick MgO was deposited by thermal evaporation. The top metal film was made from Ni coated with Au. The layers were evaporated using a shadow mask with a line width of 50 μm and thickness of 80 nm of Ni and 20 nm of Au respectively, in a cross-geometry structure relative to the bottom Au layer. The device was subsequently attached to a chip carrier and electrically connected by bonder. The sample was measured by 2T-cryogenics system (Cryogenics Ltd). A magnetic field of up to 0.6 T was applied perpendicular to the sample plane. The resistance of the device was measured using standard four-probe method. DC current of 0.5 mA was applied using a Keithley current source (Model 2400) and the voltage across the junction was measured using a Keithley nanovoltmeter (Model 2182A).

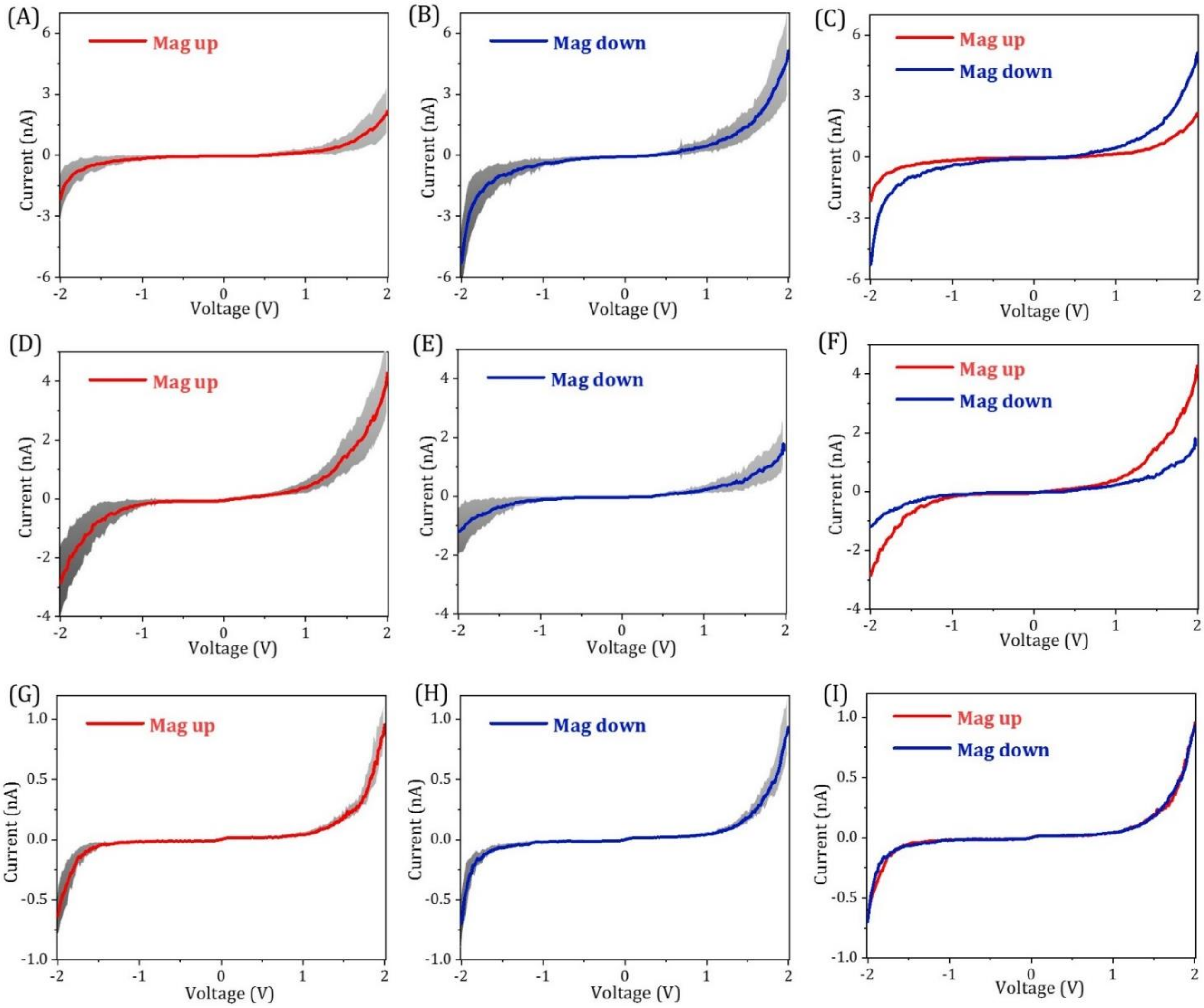

Figure S1. Spin-dependent conduction study by mc-AFM. (A-C) Current versus voltage (I-V) plots recorded for **(*S*)-1** where Ni substrate magnetized with the north pole pointing up (red) and down (blue) orientations. (D-F) I-V plots recorded for **(*R*)-1**. (G-I) I-V plots recorded for **(*n*)-1** in achiral decalin solvent. The width of the lines (in gray color) represents the standard deviation of the measurements.

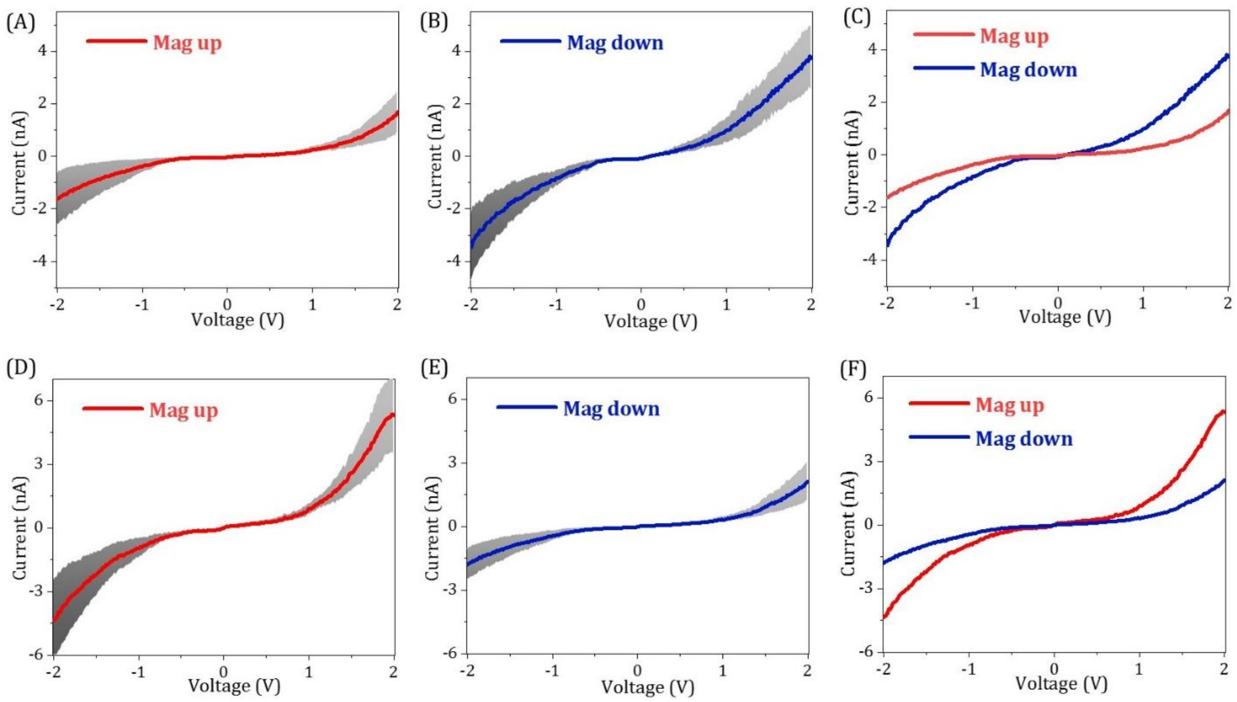

Figure S2. Spin-dependent conduction study by mCP-AFM. (A-C) Current versus voltage (I-V) plots recorded for ***(S)*-2** where Ni substrate magnetized with the north pole pointing up (red) and down (blue) orientations. (D-F) I-V plots recorded for ***(R)*-2**. The width of the lines (in gray color) represents the standard deviation of the measurements.

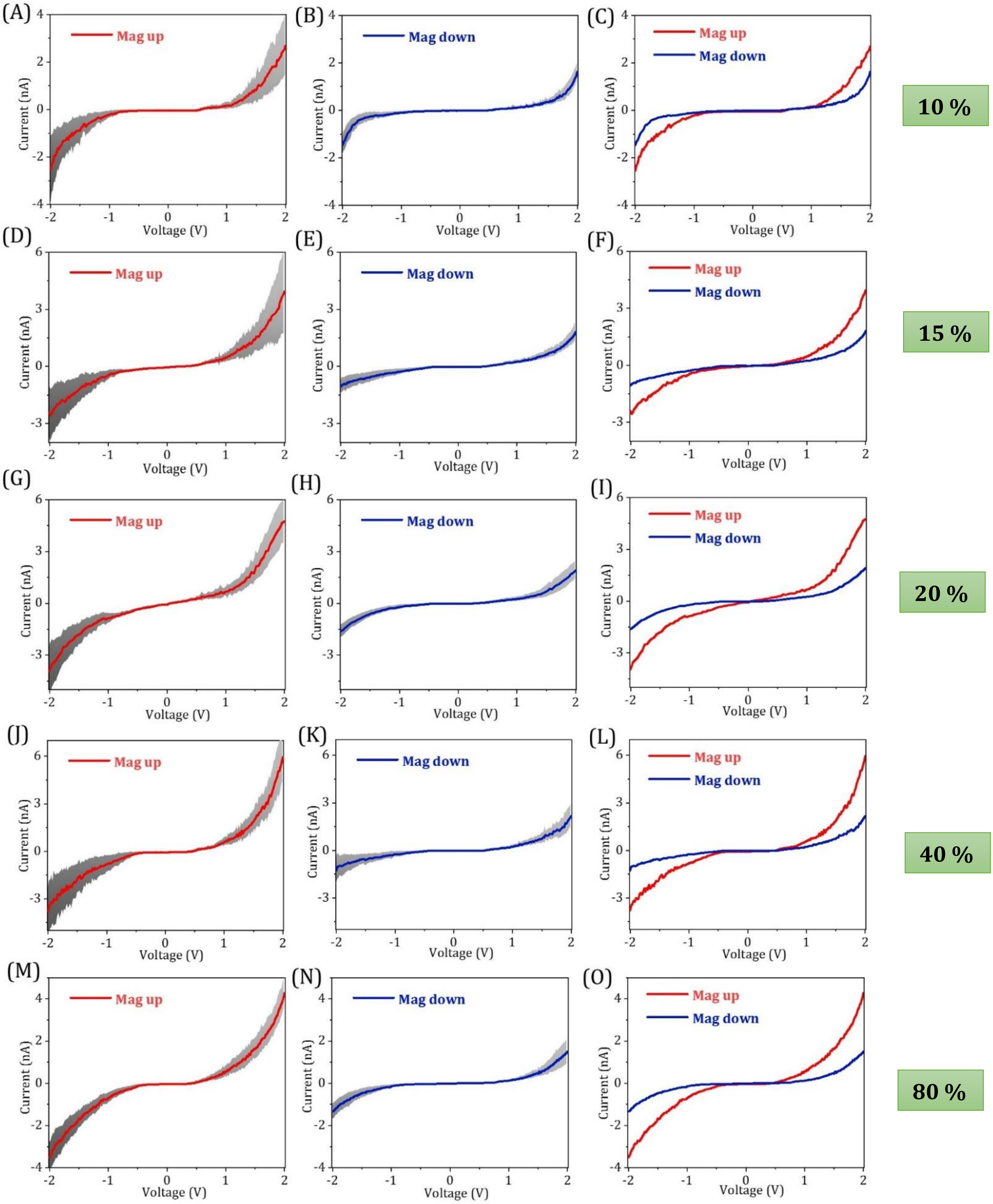

Figure S3. Spin-dependent conduction study of ***n*-1** for different % *ee* of (*S*)-ClMeBu. (A-C) I-V plots of ***n*-1** for 10% *ee* of (*S*)-ClMeBu solvent where Ni substrate magnetized with the north pole pointing up (red) and down (blue) orientations. (D-F) I-V plots of n-1 for 15% ee of (*S*)-

ClMeBu solvent. (G-I) I-V plots of n-1 for 20% ee of (*S*)-ClMeBu. (J-L) I-V plots of n-1 for 40% ee of (*S*)-ClMeBu solvent. (M-O) I-V plots of n-1 for 80% ee of (*S*)-ClMeBu. The width of the lines (in gray color) represents the standard deviation of the measurements.

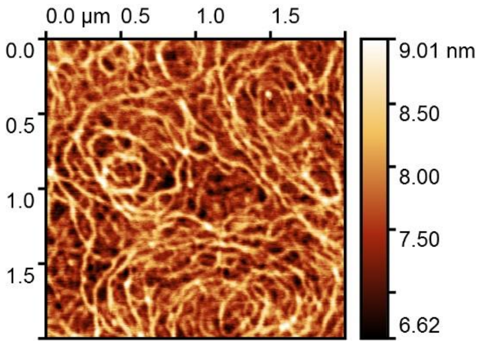

Figure S4. High-resolution AFM image of the supramolecular structures obtained from **(*R*)-1.**

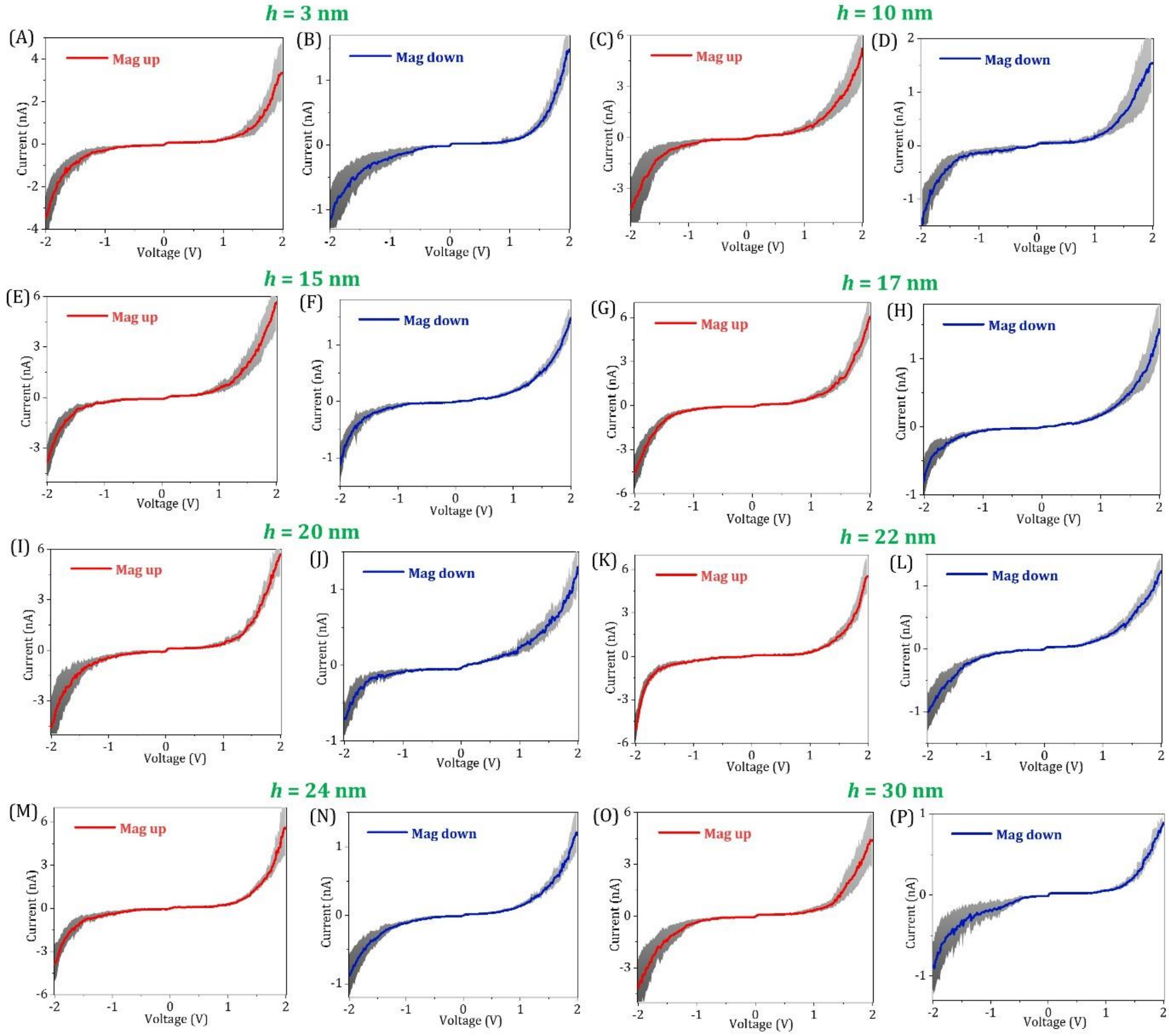

Figure S5. The film-thickness dependence (*h*) of spin polarization of **(*S*)-1**. (A-B) Current versus voltage (I-V) plots recorded for **(*S*)-1** with 3 nm of thickness. (C-D) I-V plots recorded for **(*S*)-1** with 10 nm of thickness. (E-F) I-V plots recorded with 15 nm of thickness. (G-H) I-V plots

recorded with 17 nm of thickness. (I-J) I-V plots recorded with 20 nm of thickness. (K-L) I-V plots recorded with 22 nm of thickness. (M-N) I-V plots recorded with 24 nm of thickness. (O-P) I-V plots recorded with 30 nm of thickness. In all cases, the Ni substrate were magnetized with the north pole pointing up (red) and down (blue) orientations. The width of the lines (in gray color) represents the standard deviation of the measurements.

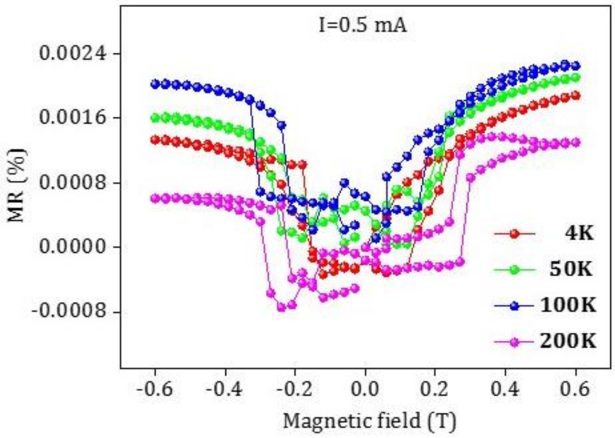

Figure S6. Magnetoresistance curves for **1** molecule in achiral solvent as a function of magnetic field between -0.6 and 0.6 T at different temperatures. The measurements were performed at a constant current of 0.5 mA.

4. Spectroscopic characterization

4.1. Solution Based Spectroscopic Characterization

UV spectra of 1 and 2 in (*S*)-CldMeOct and decaline (achiral)

Figure S7 shows the absorption profiles of 75 µM solutions of **(*S*)-1**, **(*S*)-2** and **1** in decaline (achiral) at room temperature. Independent of the solvent choice, an absorption maximum at 262 nm can be observed. This has been previously assigned to the absorption maximum of the polymeric species.[3] The high resemblance in shape between **(*S*)-1**/ **(*S*)-2** and **1** in decaline points towards similar arrangements of the chromophores in solution. Since the CD of **(*S*)-1** and **(*S*)-2** express helical arrangement while **1** in decaline is CD silent (see main text figure 1D), it can be assumed based on the provided UV and CD spectra that **1** in decaline adapts a racemic helical conformation.

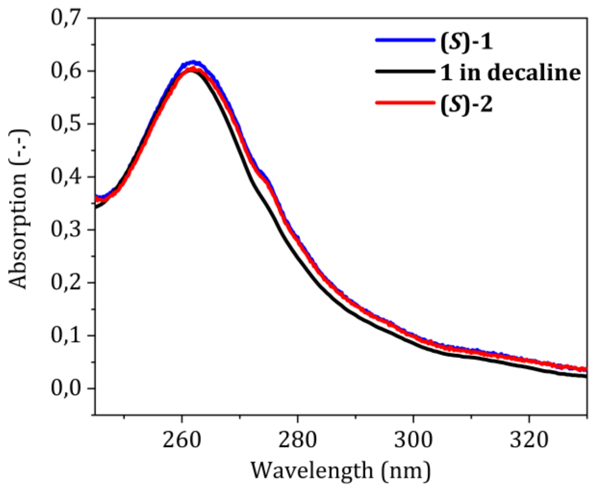

Figure S7. UV-spectra of **(*S*)-1**, **(*S*)-2** and **1** in decaline (c = 75 μM, d = 1 mm, 20 °C).

Full CD spectra of 1 in (*S*)-ClMeBu of different enantiomeric excess (*ee*)

Figure S8 shows the full CD spectra of **1** in ClMeBu of different enantiomeric excess (*ee*). A non-linear increase of the helical excess can be observed with increasing the *ee* of the solvent mixtures from 0% *ee* to 40% *ee*. At 40% *ee* the system reaches full amplification of asymmetry. Note that after mixing the stock solutions of 100% *ee* and 0% *ee* in the desired ratio, the samples were equilibrated for 1h at 70 °C since equilibration at room temperature is rather slow.

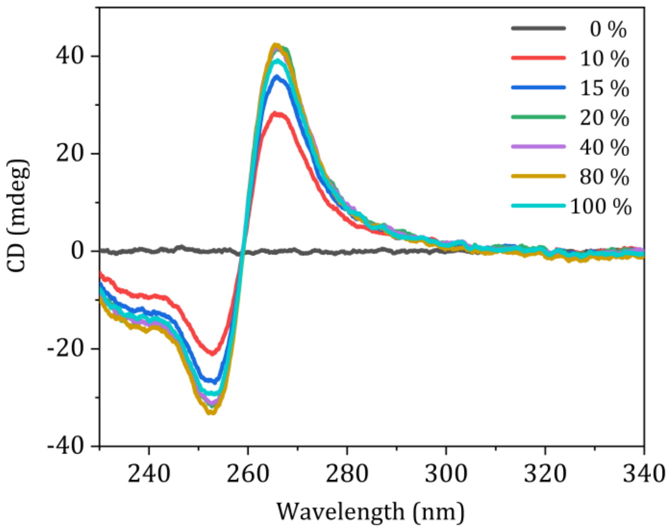

Figure S8. CD spectra of **1** for different % ee of (*S*)-ClMeBu (c = 75 μM, d = 1 mm, 20 °C).

CD-spectra of the pure solvents

The CD spectra of the pure solvents (*S*)-CldMeOct, (*R*)-CldMeOct and (*S*)-ClMeBu in Figure S9 are CD silent. Therefore, there is no contribution of the chiral solvent to the CD measurements of **1** and **2** in the spectroscopic window used for the measurements.

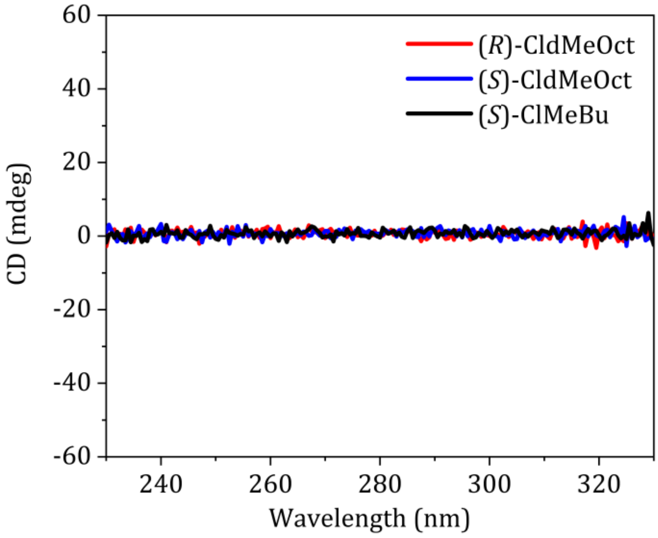

Figure S9. CD spectra of (*S*)-CldMeOct, (*R*)-CldMeOct and (*S*)-ClMeBu (d = 1 mm, 20 °C).

4.2. Solid state UV and CD characterization

Figure S10 shows the average thin film UV and CD of **1** in 100% *ee* and 0% *ee* ClMeBu. Each measurement represents the average of eight subsequent measurements of front (sample pointing towards light source) and backside (sample pointing towards detector) with a rotation around the optical axis of 0°, 120°, 240° and 360° to reveal true CD without contributions of linear dichroism and linear birefringence. The individual measurements are represented in Figure S11. While the CD spectrum is mostly invariant, the UV absorption profile shows different intensities. This could result from different degrees of scattering or variations in sample thickness upon rotation of the sample. Please note that no specific aperture for sample rotation was available and slight differences in the positioning in the optical path of the instrument could have occurred. Difficulties with deviations in the positioning can be observed by comparing exemplary the measurements for rotation of 0° and 360° (which should in theory equal the spectra of 0°).

The absorption profiles of **1** in 0% *ee* and 100% *ee* show a strong resemblance in shape ($\lambda_{max,solution}$= 262 nm) to the one observed in solution, indicating similar arrangement of the chromophores in bulk and in solution (Figure S7). Small differences in the absorption maximum for 0% *ee* and 100% *ee* of 266 nm and 270 nm respectively can be observed but might be regarded as negligible in respect to the challenging experiment. Further, the shoulder observed in solution at 311 nm appears red-shifted in bulk at 318 nm for both samples. Most strikingly, no monomer absorption[3] is obtained after removing the solvent, confirming the stability of the supramolecular polymers in bulk as already showcased by AFM (Figure 1C and Figure S4). The bisignate Cotton effect observed in solution for **1** in 100% *ee* (Figure S8) with maxima at 253 nm and 266 nm also appear in bulk but with a shift towards lower energies with maxima at 257 nm and 280 nm. The red shift of the low energy CD band is interestingly more pronounced than the one observed for the high energy CD band. Further the hardly noticeable shoulder around 315 nm in solution is more pronounced in bulk with a negligible red shift to 312 nm. The $g_{abs}(\lambda) = (CD(\lambda))/(Abs(\lambda) \times 32980)$ were calculated as 0.022 and -0.019 for 280 nm and 257 nm respectively.

Despite slight differences between spectra obtained from solution or bulk and difficulties in obtaining comparable samples, it can be clearly observed that samples spin-coated from optically

inactive solvents also result in optically inactive thin films, while spin-coating from optically active solvents results in strong CD active samples. This implies that the structures processed from optically active solvents can memorize the chiral information from the solvent even after removing the chiral stimulus. Further as evidenced by the UV spectra, no disassembly of the polymers occurs on the surface.

Please note that the samples have a high tendency to cluster/ crystallize on the surface, making it extremely challenging to obtain suitable films for spectroscopic characterization. In the case of (*S*) and (*R*)-CldMeOct, the low volatility and hence slow evaporation of the solvent did not allow to produce suitable films due to the effects mentioned before.

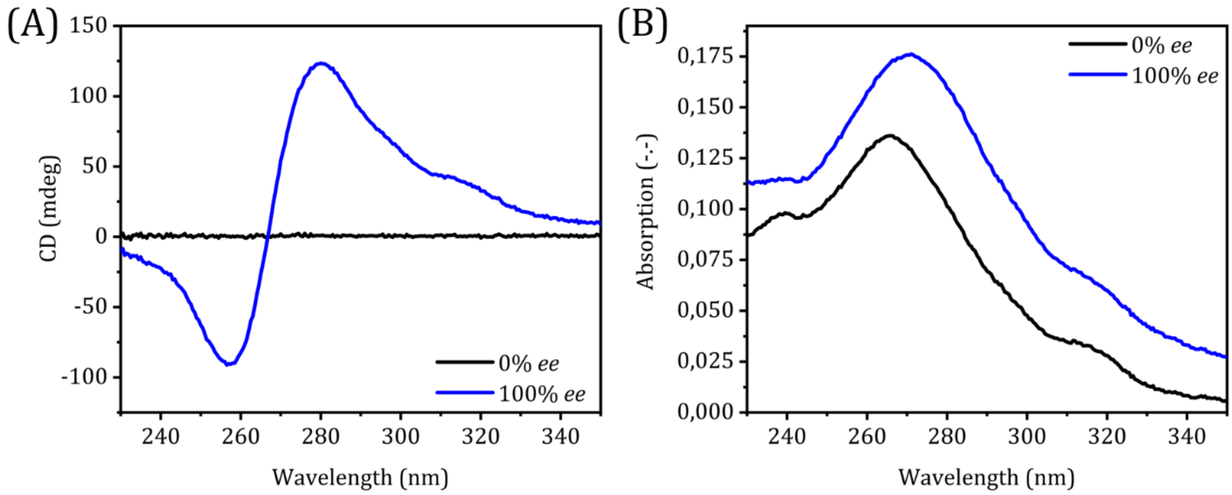

Figure S10 Averaged thin film spectra of **1** spin-coated from 100% *ee* and 0% *ee* ClMeBu on quartz glass. (A) CD (B) UV.

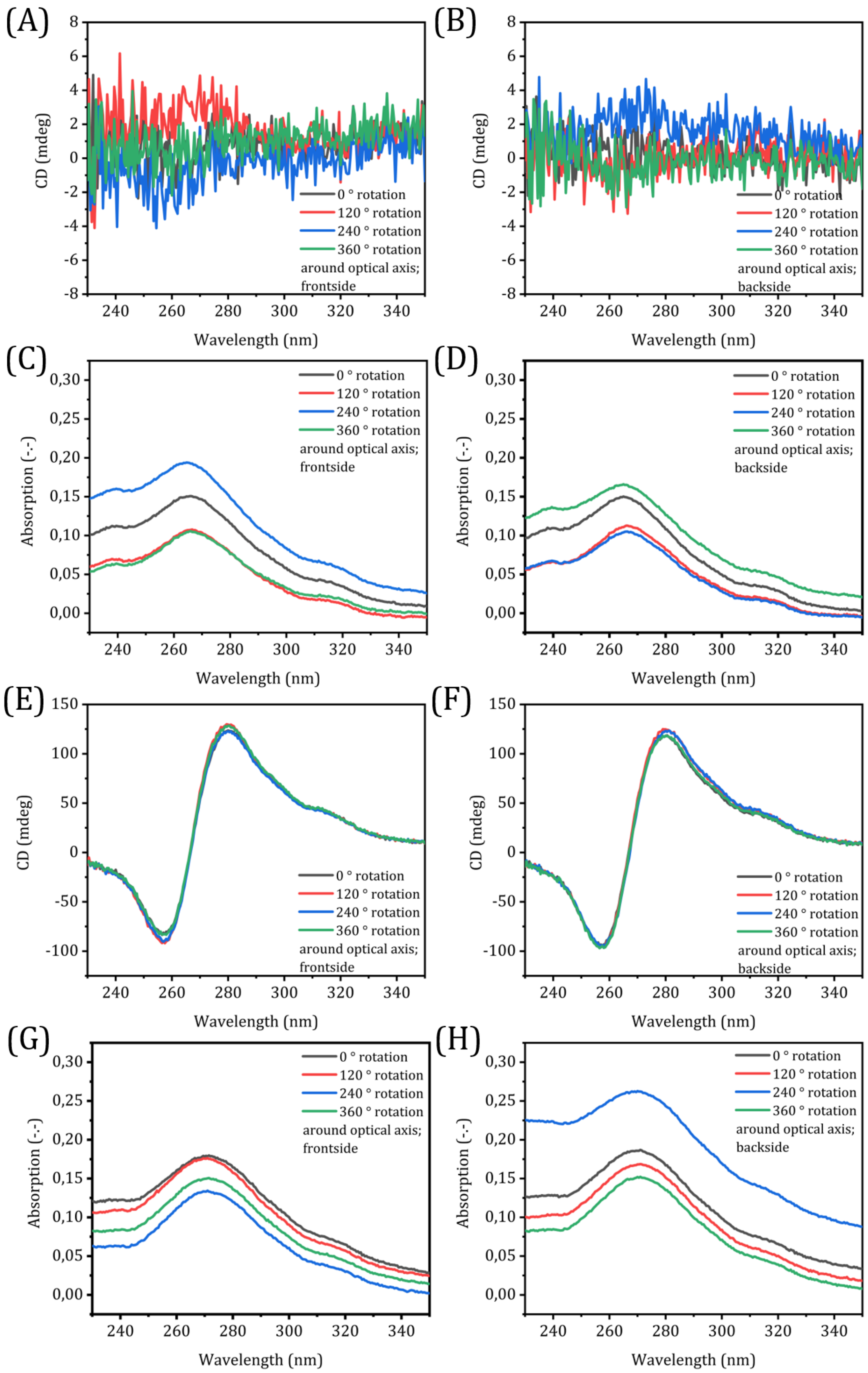

Figure S11 Frontside and backside CD and UV measurements with rotation around the optical axis of 0°, 120°, 240° and 360°. (A)/(B) CD and (C)/(D) UV spectra of **1** spin-coated from 0% *ee* ClMeBu; (E)/(F) CD and (G)/(H) UV spectra of **1** spin-coated from 100% *ee* ClMeBu.

5. Spectroscopic Characterization of Final Compounds

N[2],*N*[6],*N*[10]-Tris(octyl)triphenylene-2,6,10-tricarboxamide (5-(*n-8*))

^{1}H-NMR (400 MHz, $CDCl_3$ + TFA-*d*)

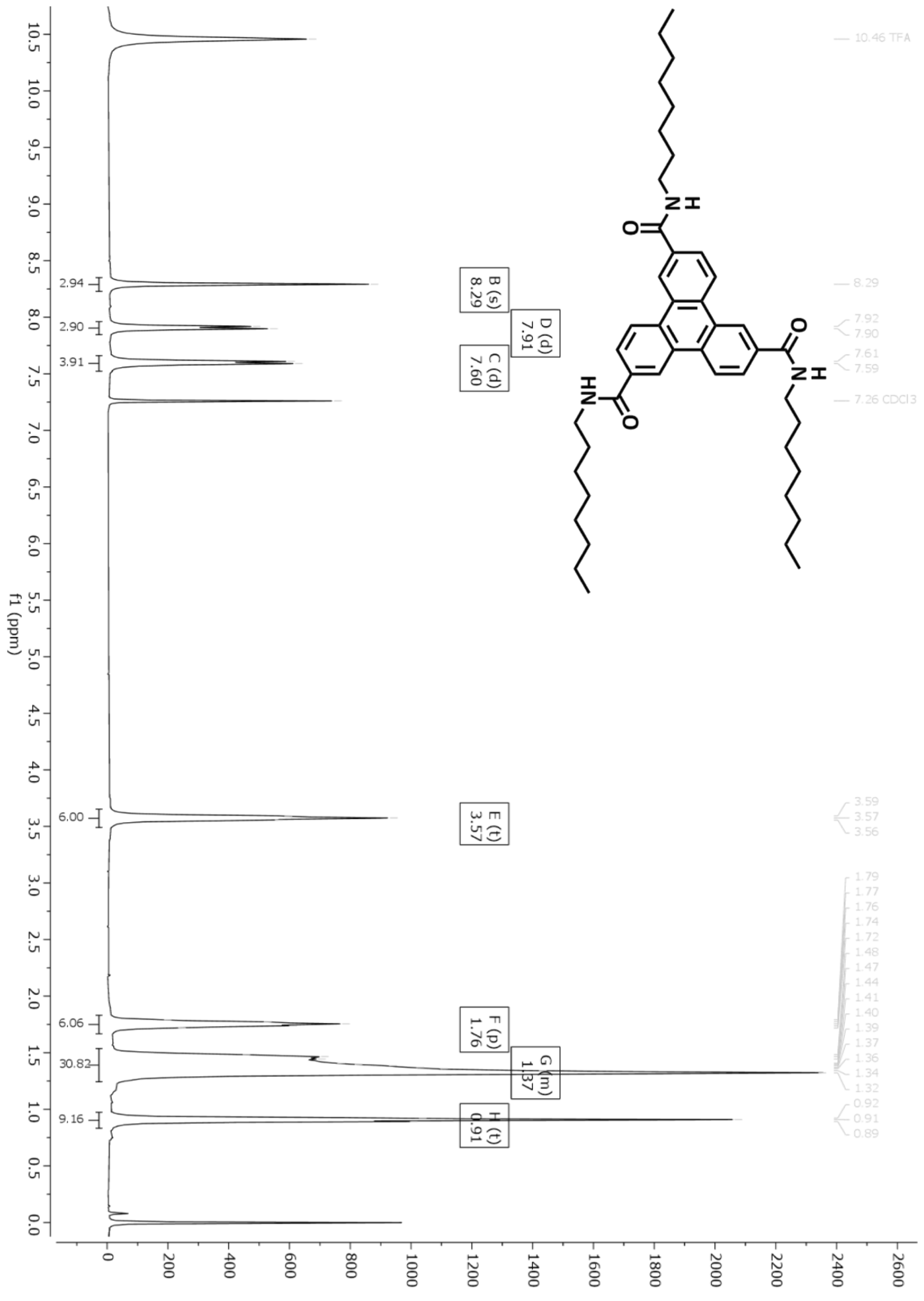

^{13}C-NMR (101 MHz, $CDCl_3$ + TFA-*d*)

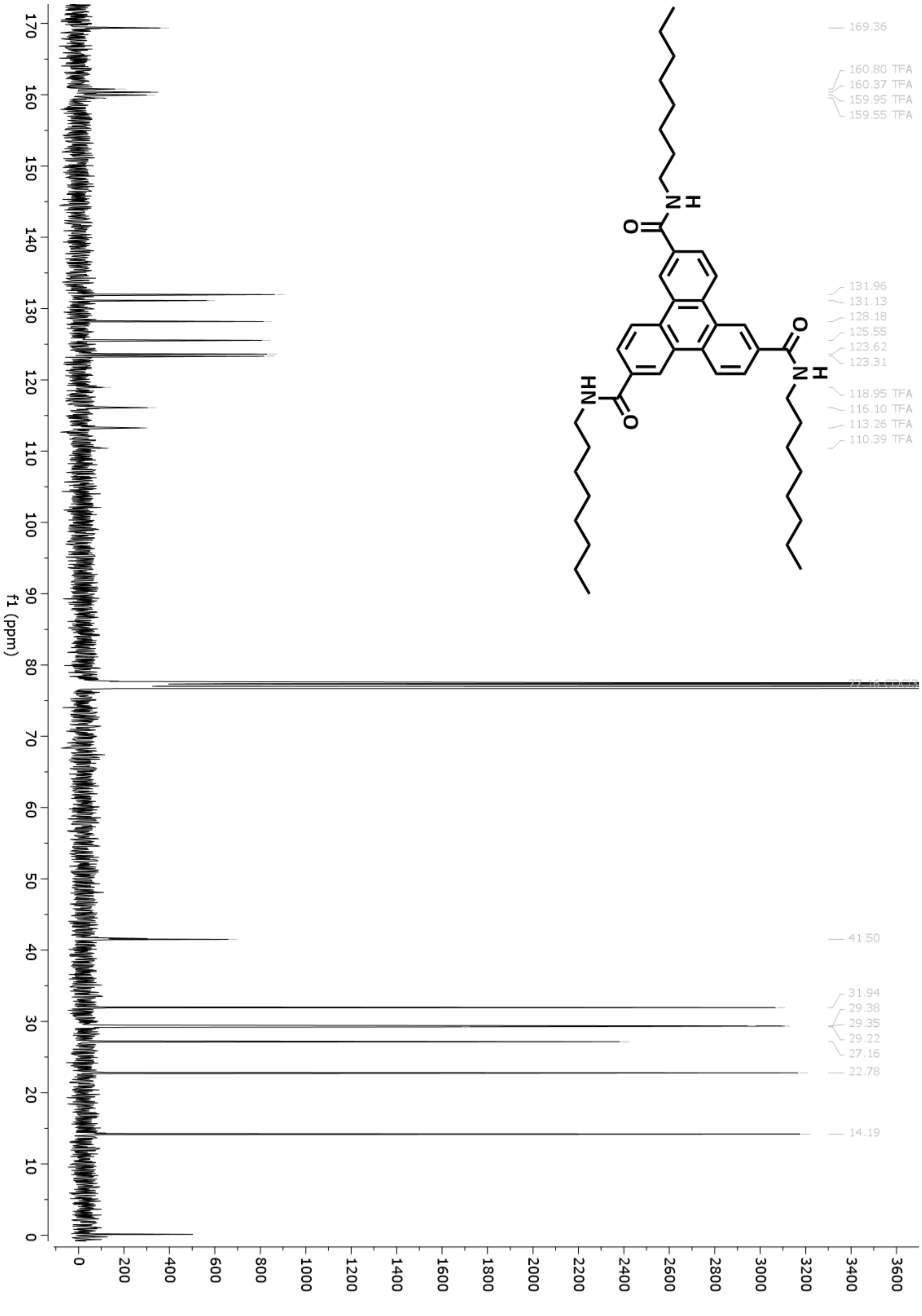

COSY

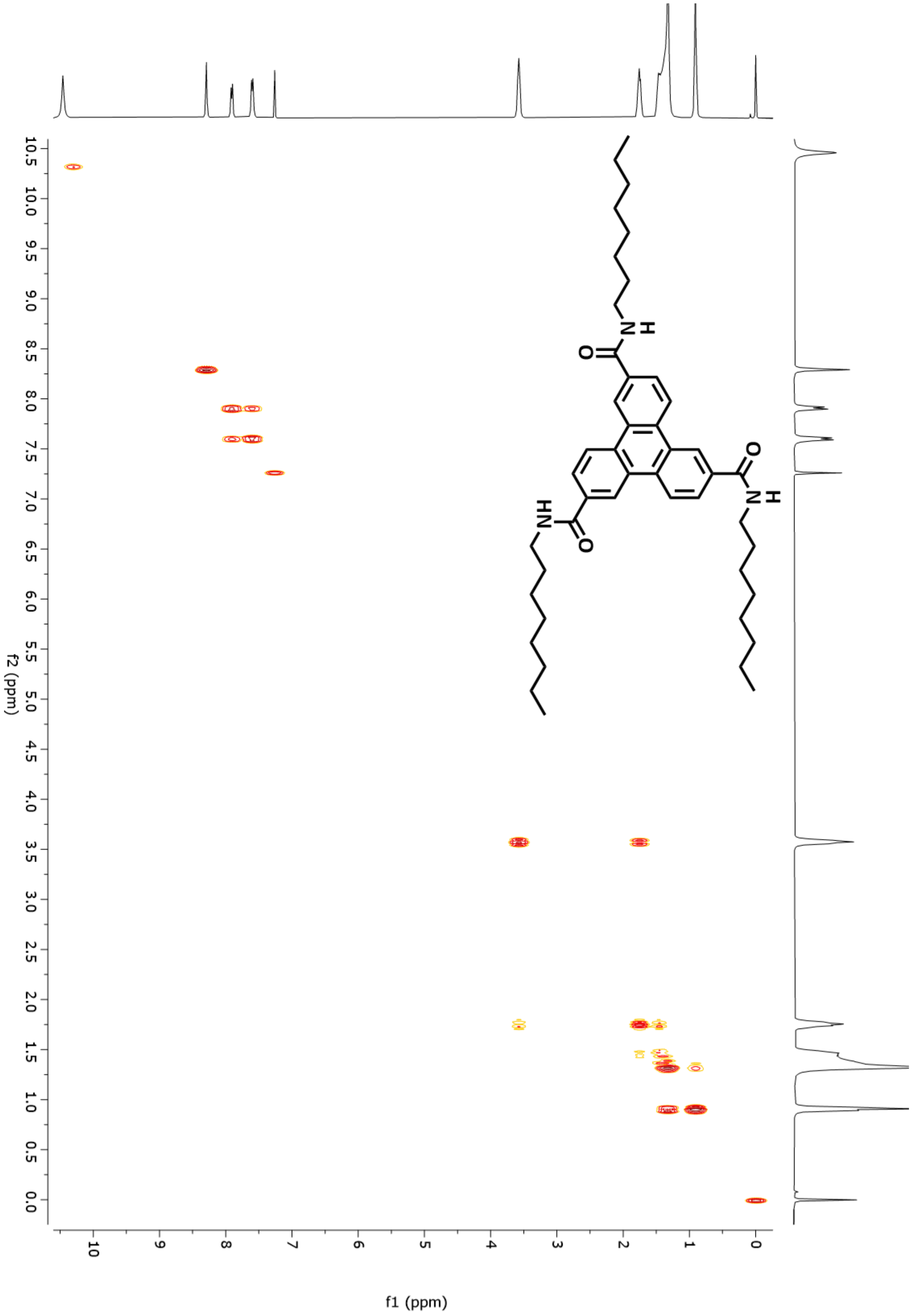

HSQC

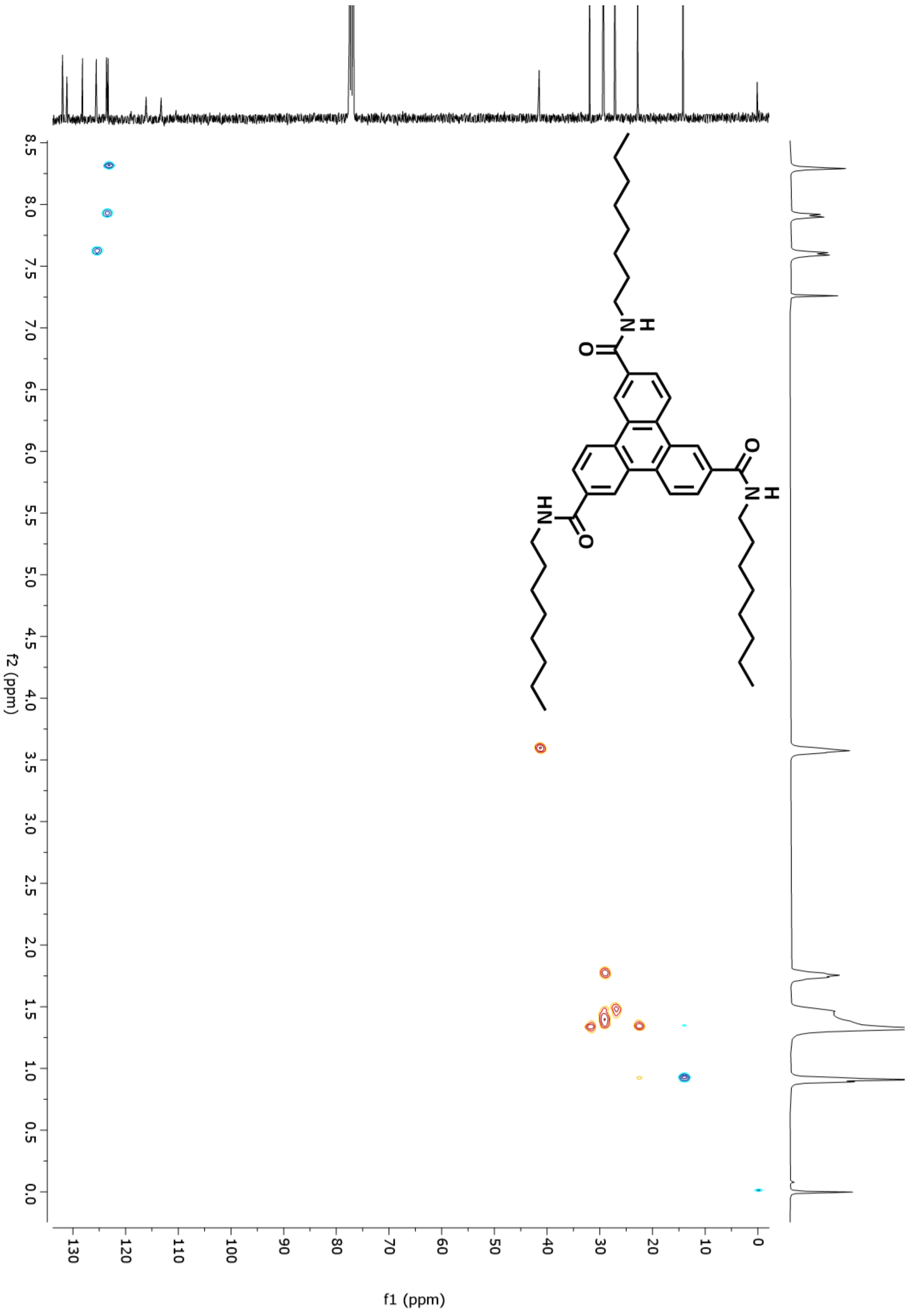

HMBC

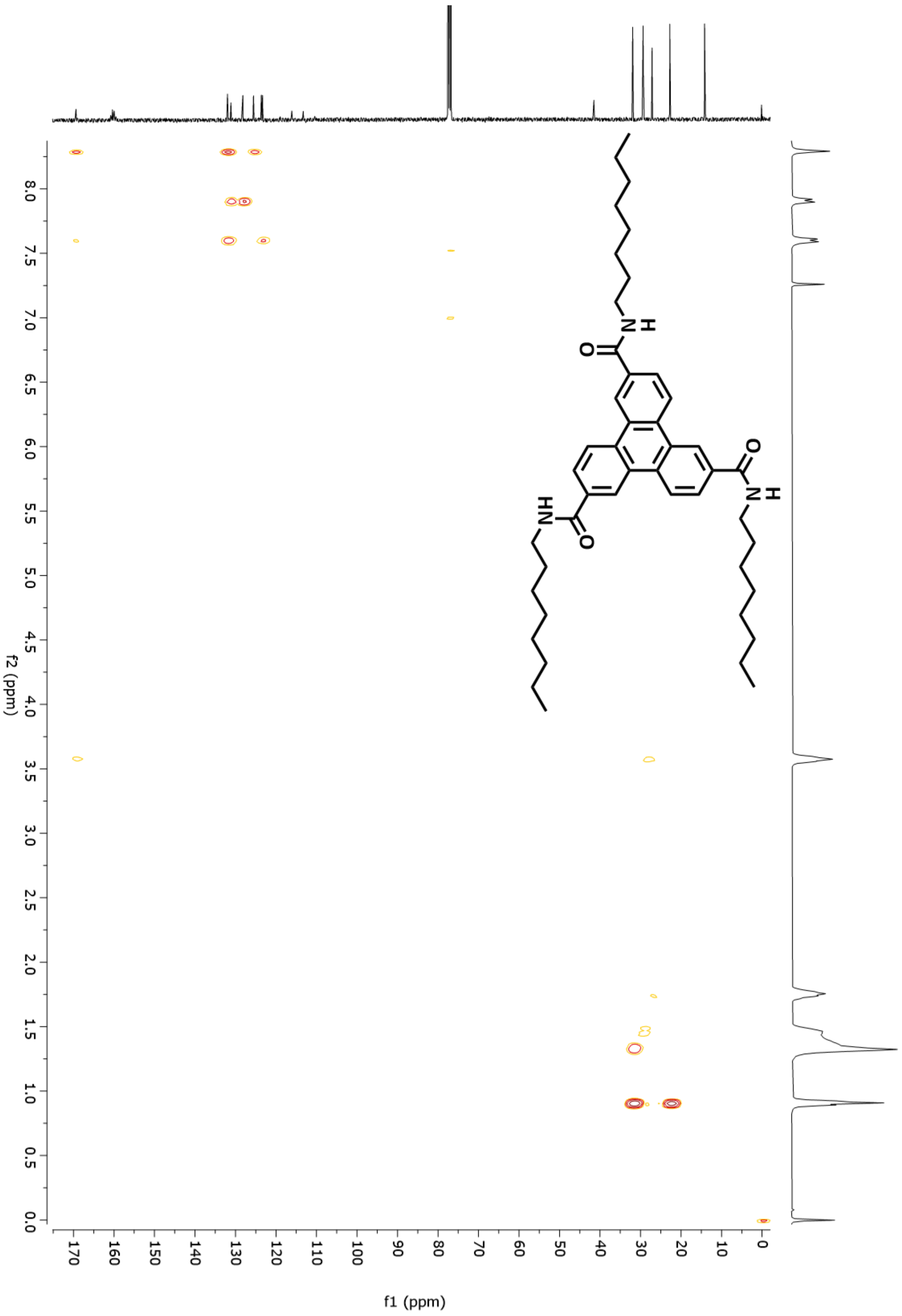

N^2,N^6,N^{10}-Tris(decyl)triphenylene-2,6,10-tricarboxamide (5-(*n*-*10*))

^{1}H-NMR (400 MHz, $CDCl_3$ + TFA-*d*)

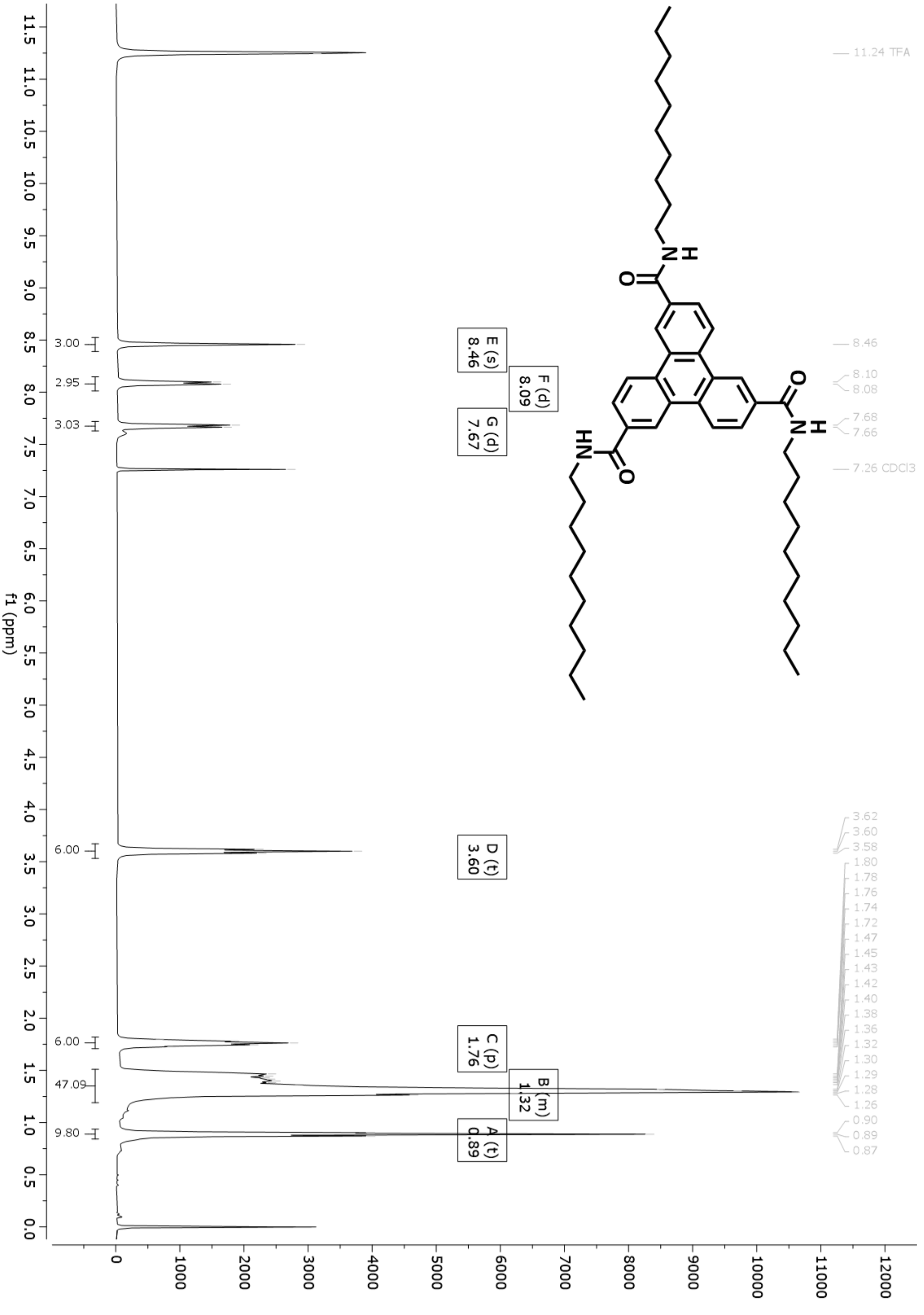

^{13}C-NMR (101 MHz, $CDCl_3$ + TFA-*d*)

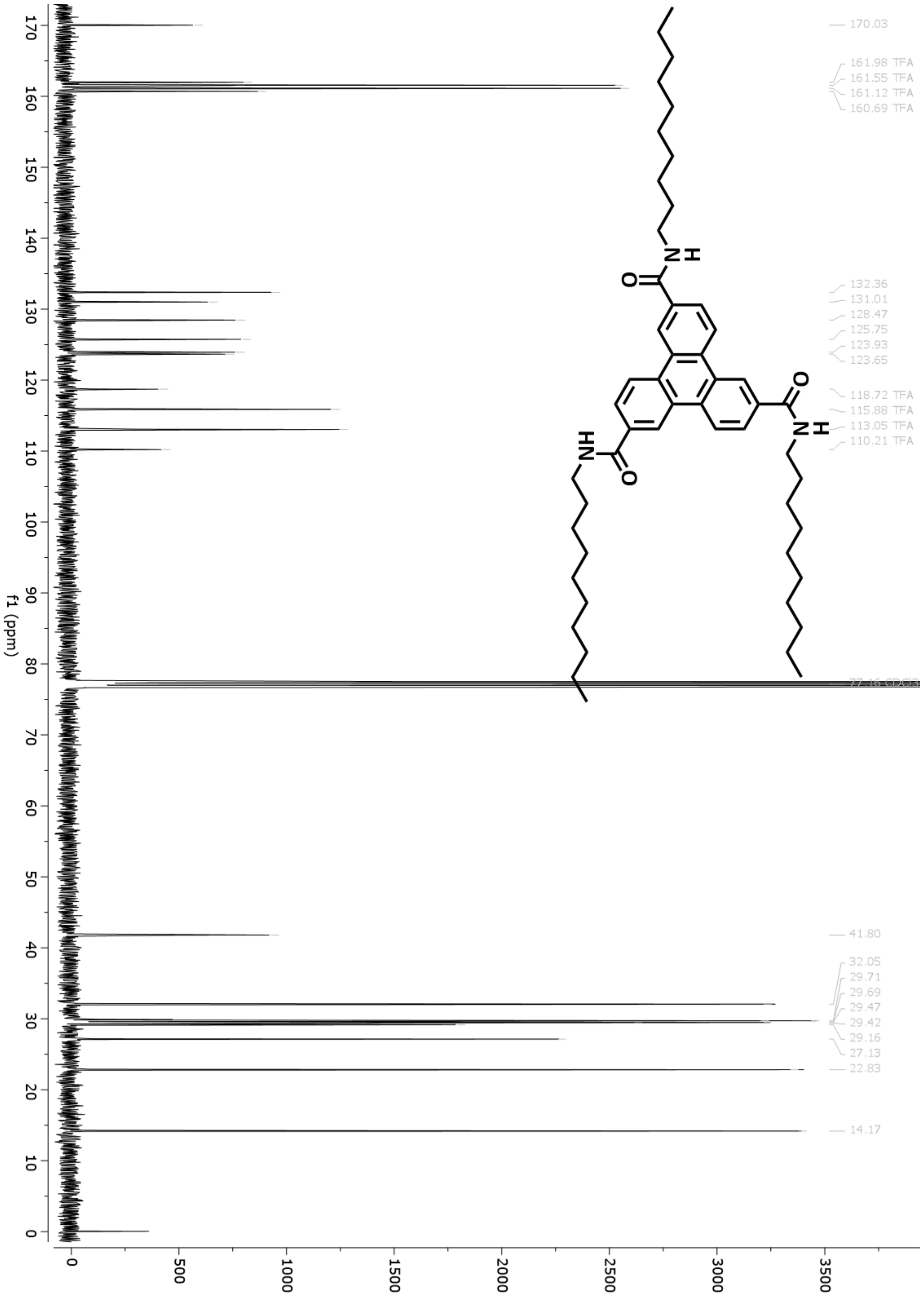

COSY

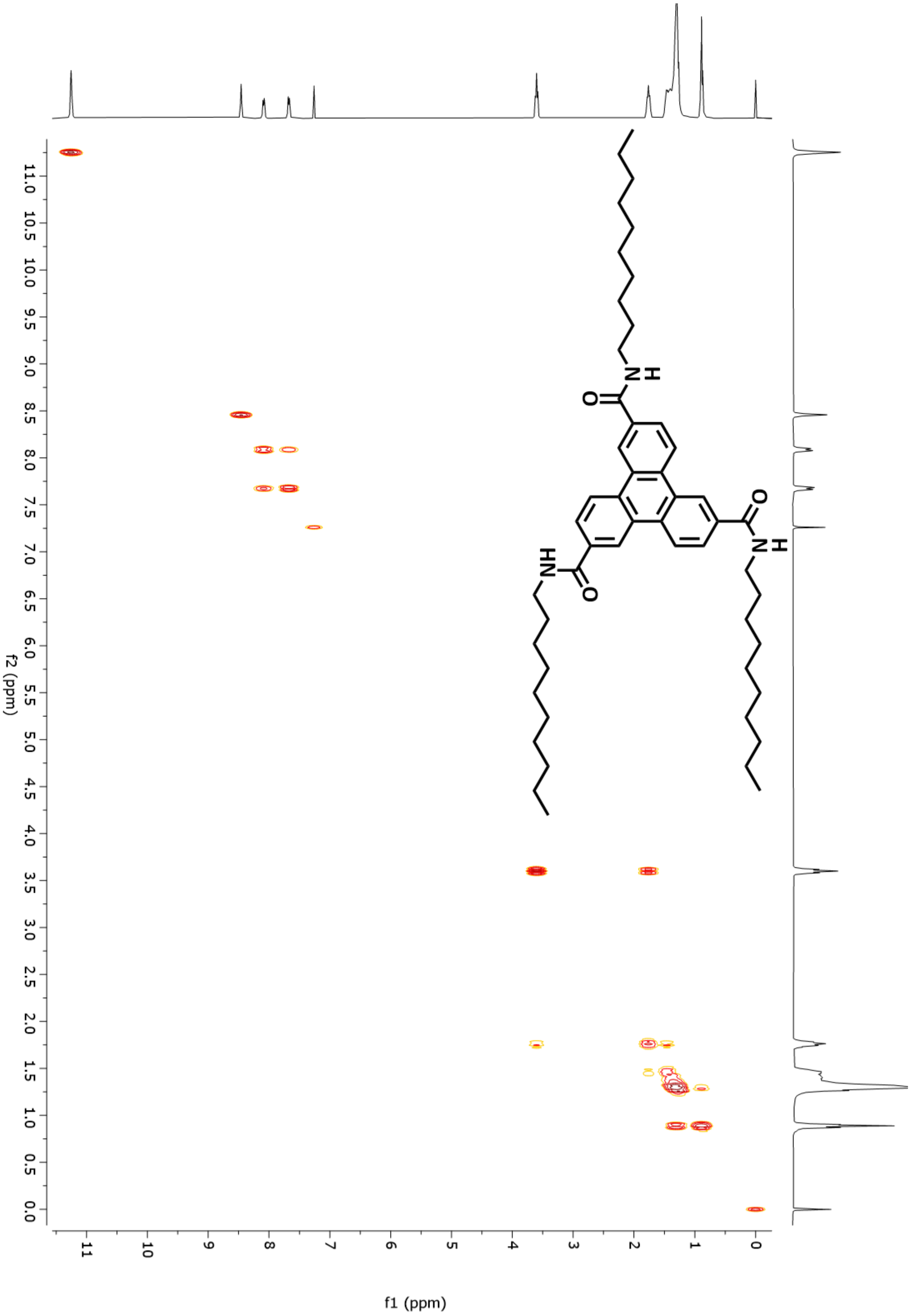

HSQC

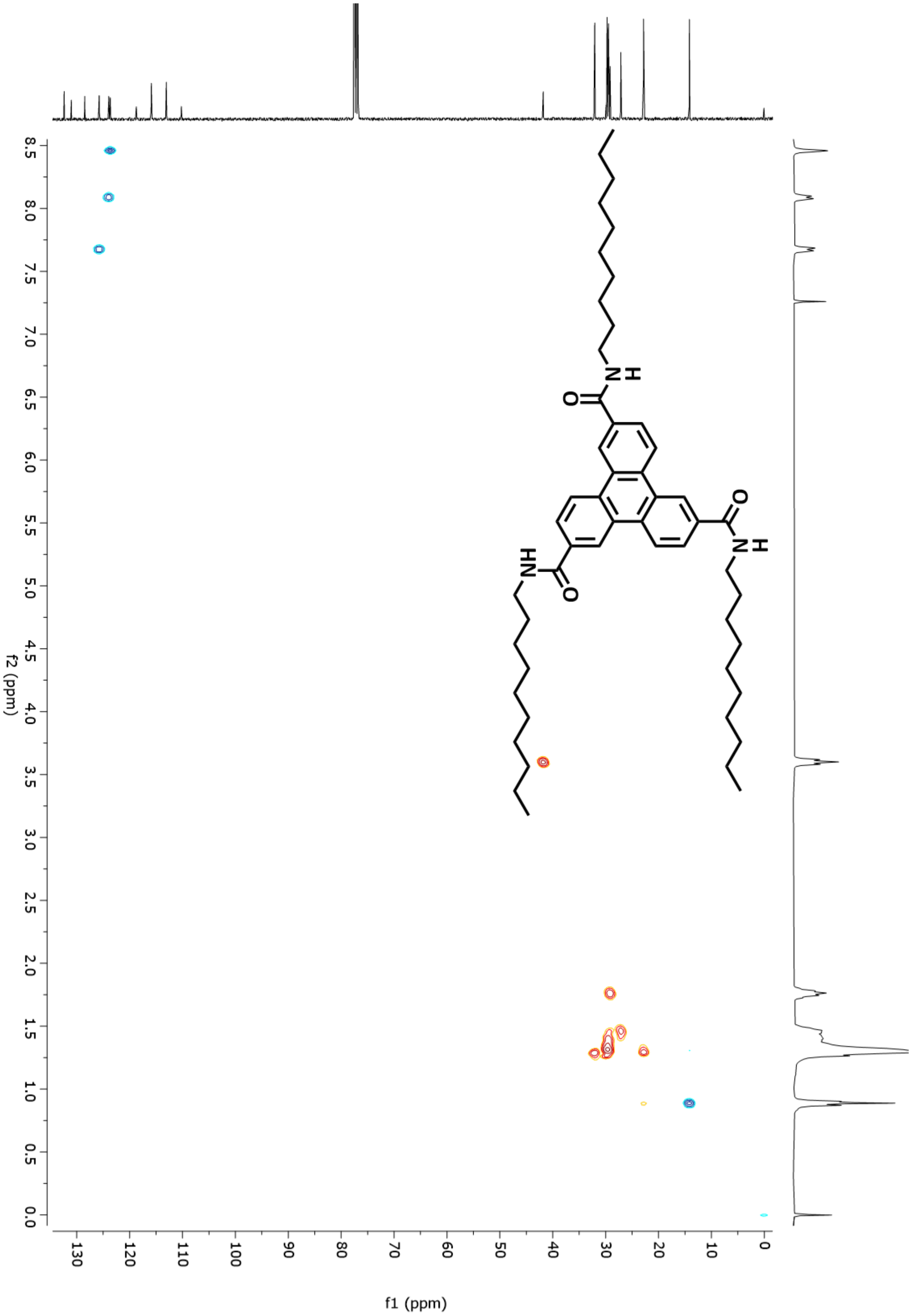

HMBC

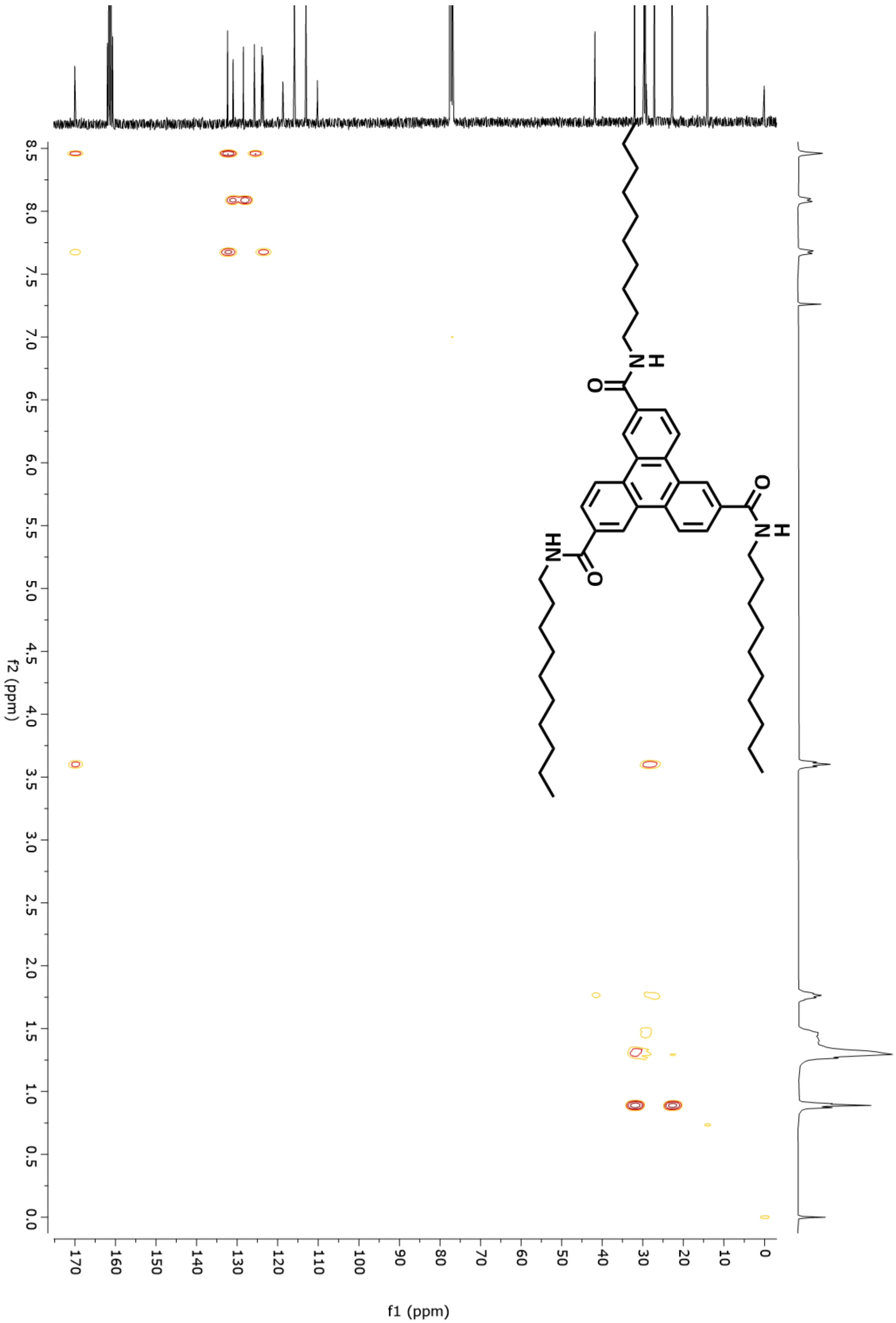

(*S*)-1-Cl-3,7-dimethyloctane ((*S*)-8)

^{1}H-NMR (400 MHz, $CDCl_3$)

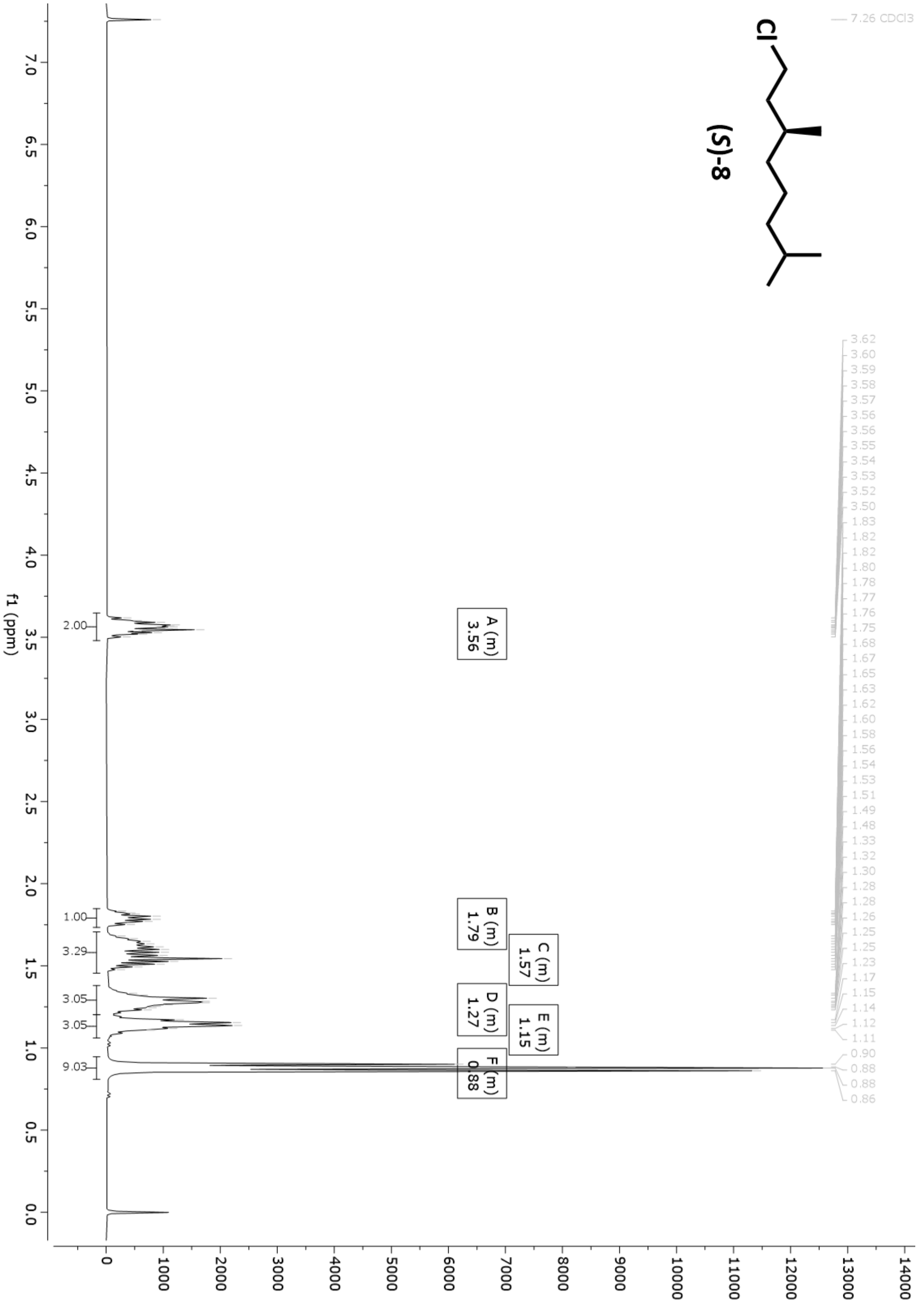

^{13}C-NMR (101 MHz, $CDCl_3$)

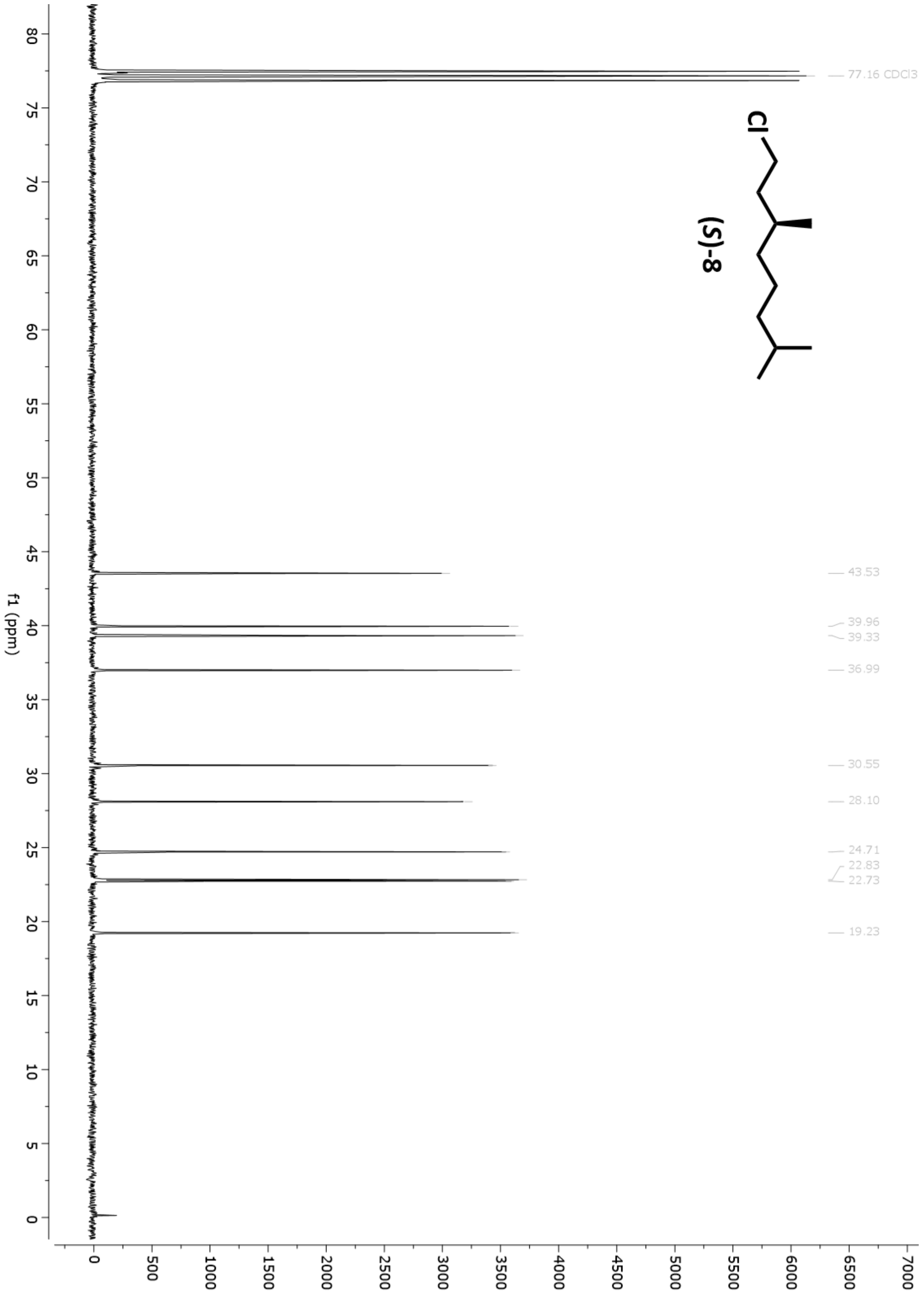

(R)-1-Cl-3,7-dimethyloctane ((R)-8)

^{1}H-NMR (400 MHz, $CDCl_3$)

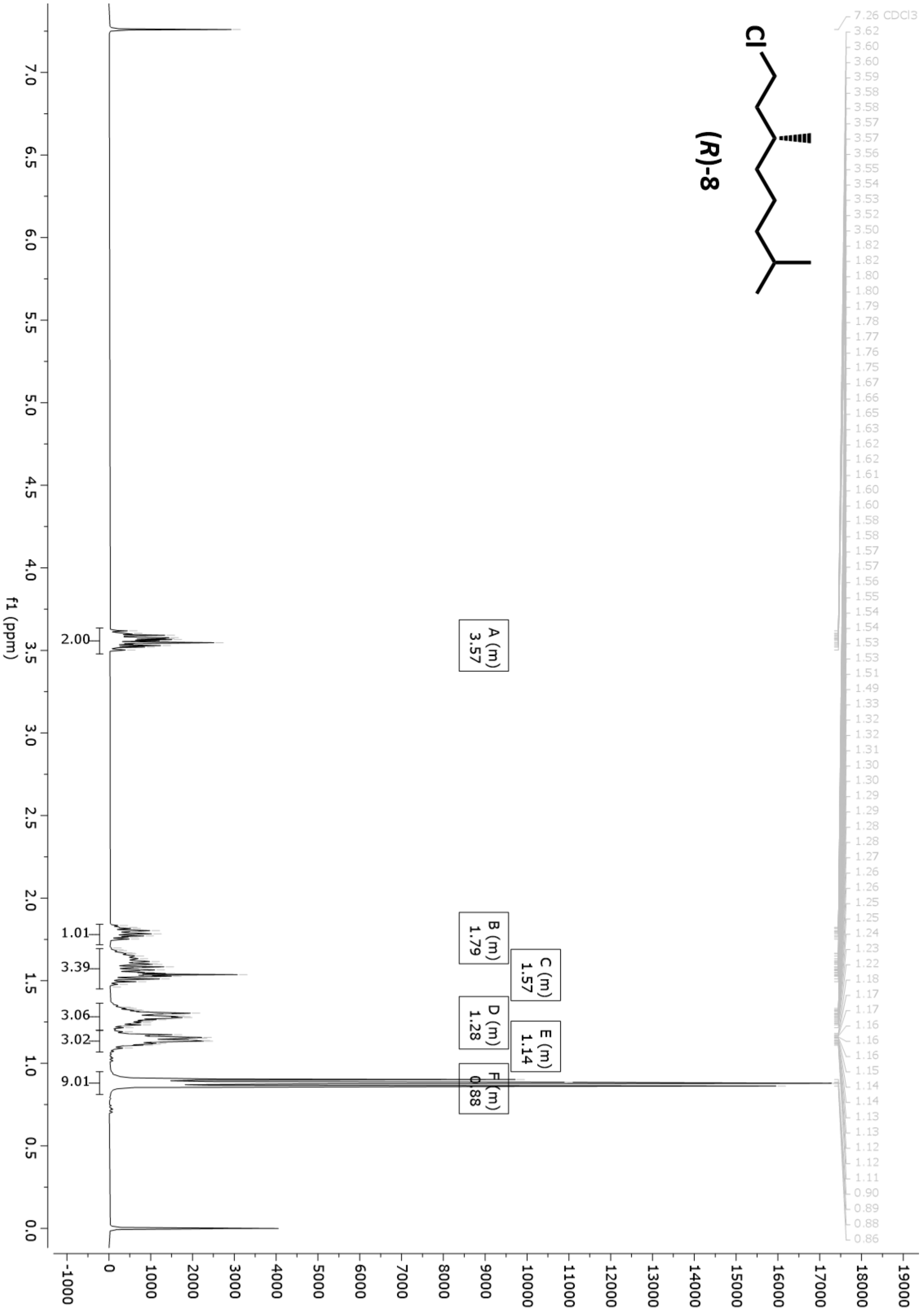

^{13}C-NMR (101 MHz, $CDCl_3$)

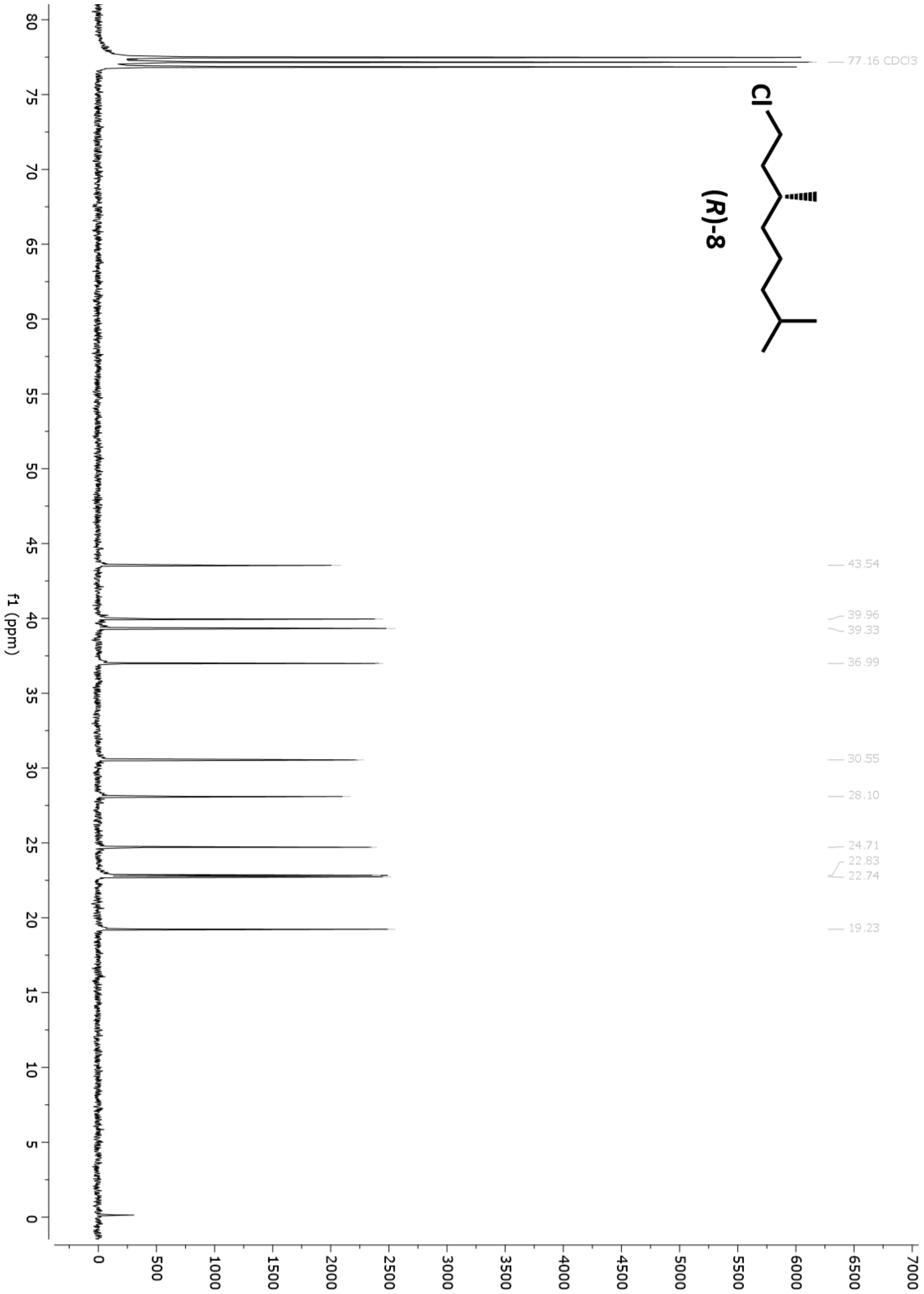

(*S*)-1-chloro-2-methylbutane ((*S*)-10)

^{1}H-NMR (400 MHz, $CDCl_3$)

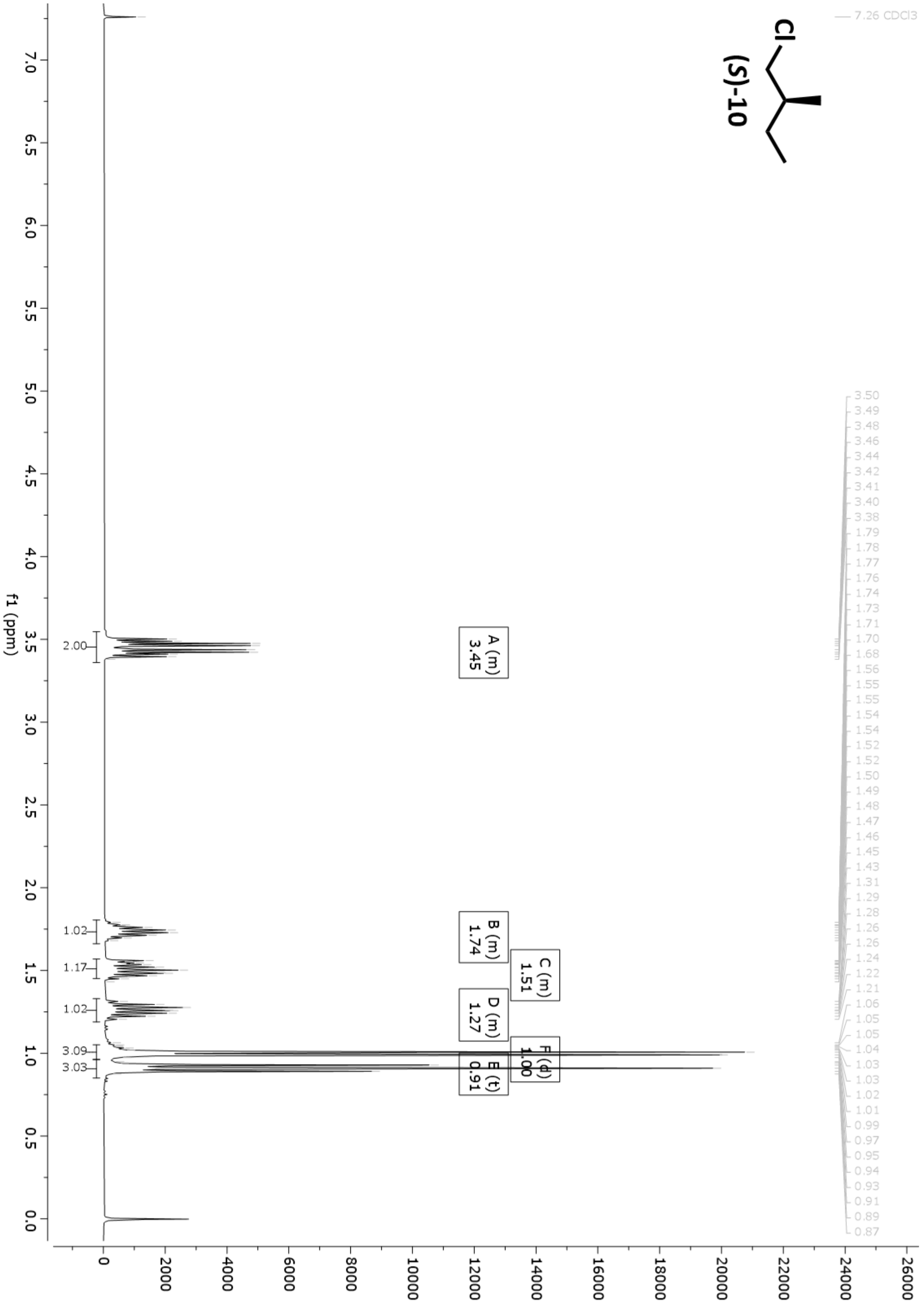

^{13}C-NMR (101 MHz, $CDCl_3$)

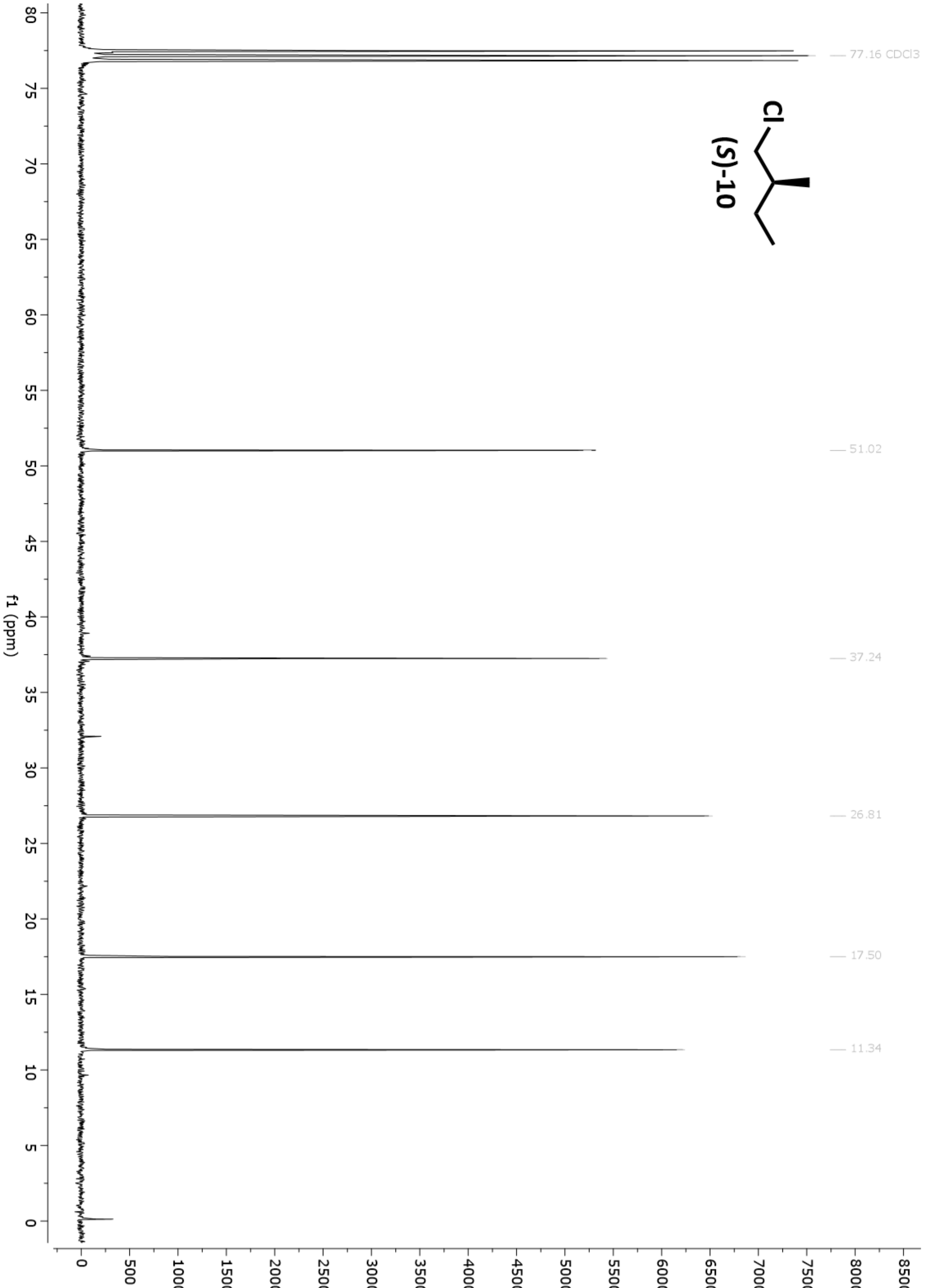

[1] Bock, H.; Rajaoarivelo, M.; Clavaguera, S.; Grelet, E. An efficient route to stable room temperature liquid-crystalline triphenylenes. *Eur. J. Org. Chem.* **2006**, 2889–2893.

[2] Shirai, H.; Amano, N.; Hashimoto, Y.; Fukui, E.; Ishii, Y.; Ogawa, M. Trisannelated Benzene Synthesis by Zirconium Halide Catalyzed Cyclohydration of Cycloalkanones. *J. Org. Chem.* **1991**, *56*, 2253–2256.

[3] Ślęczkowski, M. L.; Mabesoone, M. F. J.; Ślęczkowski, P.; Palmans, A. R. A.; Meijer, E. W. Competition between chiral solvents and chiral monomers in the helical bias of supramolecular polymers. *Nat. Chem.* **2020**, *13*, 200–207.
